# Tumor-mediated immunosuppression and cytokine spreading affects the relation between EMT and PD-L1 status

**DOI:** 10.3389/fimmu.2023.1219669

**Published:** 2023-08-10

**Authors:** Carlijn M. Lems, Gerhard A. Burger, Joost B. Beltman

**Affiliations:** Division of Drug Discovery and Safety, Leiden Academic Centre for Drug Research, Leiden University, Leiden, Netherlands

**Keywords:** epithelial-mesenchymal transition (EMT), PD-L1, immunoevasion, ordinary differential equations, cellular Potts model

## Abstract

Epithelial-mesenchymal transition (EMT) and immune resistance mediated by Programmed Death-Ligand 1 (PD-L1) upregulation are established drivers of tumor progression. Their bi-directional crosstalk has been proposed to facilitate tumor immunoevasion, yet the impact of immunosuppression and spatial heterogeneity on the interplay between these processes remains to be characterized. Here we study the role of these factors using mathematical and spatial models. We first designed models incorporating immunosuppressive effects on T cells mediated *via* PD-L1 and the EMT-inducing cytokine Transforming Growth Factor beta (TGFβ). Our models predict that PD-L1-mediated immunosuppression merely reduces the difference in PD-L1 levels between EMT states, while TGFβ-mediated suppression also causes PD-L1 expression to correlate negatively with TGFβ within each EMT phenotype. We subsequently embedded the models in multi-scale spatial simulations to explicitly describe heterogeneity in cytokine levels and intratumoral heterogeneity. Our multi-scale models show that Interferon gamma (IFNγ)-induced partial EMT of a tumor cell subpopulation can provide some, albeit limited protection to bystander tumor cells. Moreover, our simulations show that the true relationship between EMT status and PD-L1 expression may be hidden at the population level, highlighting the importance of studying EMT and PD-L1 status at the single-cell level. Our findings deepen the understanding of the interactions between EMT and the immune response, which is crucial for developing novel diagnostics and therapeutics for cancer patients.

## 1 Introduction

Activating invasion and metastasis, and avoiding immune destruction are core hallmarks of cancer, i.e., acquired capabilities that are crucial for the formation of malignant tumors (1). A comprehensive understanding of the interplay between these hallmarks is imperative for developing novel diagnostic and therapeutic approaches. Still, few studies to date have focused on the interaction between metastatic dissemination and immunoevasion, and hence its biological basis remains in large part unexplored.

Epithelial-mesenchymal transition (EMT), a process during which cells transition from an adhesive epithelial to a motile mesenchymal phenotype (2), is of critical importance for invasion and metastasis (reviewed in (3–5)). This phenomenon is increasingly referred to as epithelial-mesenchymal plasticity (EMP), because emerging evidence suggests that this transition is often incomplete, resulting in the manifestation of intermediate epithelial/mesenchymal (E/M) phenotypes (6). Such partial EMT programs in particular are associated with enhanced metastatic dissemination as well as therapy resistance (7, and reviewed in (8)). Moreover, EMT has been proposed to facilitate tumor immune escape (reviewed in 9).

A well-established mechanism through which cancer cells acquire immune resistance involves co-opting immune checkpoint pathways (10). Under normal physiological conditions, these pathways are pivotal for modulating the immune response and maintaining self-tolerance. As a case in point, tumor cells often upregulate the immune checkpoint protein Programmed Death-Ligand 1 (PD-L1) (11), either in response to inflammatory cytokines, such as Interferon gamma (IFNγ), or through constitutive oncogenic signaling (10). Interaction of PD-L1 with its receptor Programmed Death-1 (PD-1) on the membrane of T cells suppresses the survival, proliferation, and effector functions of these cells, including their cytokine release (12).

The literature reports numerous links between immunoevasion mediated by PD-L1 and EMT (reviewed in 13). One mechanism proposedly underlying the crosstalk between EMT and PD-L1-mediated immune resistance is that PD-L1 is post-transcriptionally regulated by the microRNA-200 (miR-200)–Zinc Finger E-Box Binding Homeobox 1 (ZEB1) axis (14–16), which is part of the ‘core’ EMT regulatory machinery (6). The binding of miR-200 to PD-L1 mRNA inhibits translation of the checkpoint ligand, and such binding can generally promote degradation of the miRNA–mRNA complex (17, 18). To investigate this mechanism, we recently presented a mathematical model connecting a model for the core EMT network to a model for IFNγ-induced PD-L1 expression (19), considering mutual inhibitory feedback between miR-200 and PD-L1. Model analysis showed that this interaction gives rise to tristability in PD-L1 levels, with a mesenchymal state corresponding with high PD-L1 expression, an epithelial state with low PD-L1 expression, and an E/M state with intermediate (albeit still relatively low) PD-L1 expression. Stimulation with IFNγ further amplifies the difference in PD-L1 expression between the stable EMT states. Furthermore, the bi-directional crosstalk between miR-200 and PD-L1 reduces the amount of inducing signal required to undergo EMT in the presence of IFNγ.

Despite displaying interesting dynamics relevant for tumor progression, our prior model of EMT–PD-L1 dynamics (19) did not take into account several mechanisms and factors affecting EMT and PD-L1 expression. First, an important missing mechanism was the negative feedback of PD-L1 on the IFNγ secretion of T cells, which results from the PD-L1–PD-1 interaction (20). Second, our prior model did not explicitly describe Transforming Growth Factor beta (TGFβ) as an EMT-inducing signal, and as a central player in tumor immune evasion (reviewed in 21). Of particular relevance here is the ability of TGFβ to inhibit IFNγ release both directly and indirectly by inhibiting T cell proliferation and differentiation. Third, our regulatory EMT–PD-L1 network model did not consider the potential role of spatial effects, such as the spatiotemporal and potentially localized spreading of cytokines within the tumor microenvironment (TME). Fourth, the model described the behavior of an average tumor cell and therefore did not account for intratumoral heterogeneity, which was recently demonstrated to contribute to resistance to PD-(L)1 blockade (22).

In the present study, we extended the model presented by Burger et al. (19) to explore the role of immunosuppression through PD-L1 or TGFβ, and of intratumoral heterogeneity on the crosstalk between EMT and PD-L1 expression. Analysis of our models with immunosuppression shows that negative feedback of PD-L1 on IFNγ only decreases the difference in PD-L1 expression between EMT phenotypes, whereas TGFβ-mediated IFNγ inhibition gives rise to a negative correlation between TGFβ and PD-L1 levels within EMT phenotypes. By subsequently embedding the above networks in multi-scale cell-based spatial simulations with cytokine spreading and intratumoral heterogeneity, we show that partial EMT of a tumor cell subset induced by IFNγ offers bystander tumor cells limited protection from IFNγ. Moreover, we demonstrate that a study at the cell population level may hide the underlying relation between PD-L1 expression and EMT status. Overall, our analysis illustrates how tumor-mediated immunosuppression and cytokine spreading can affect the complex relationship between EMT and PD-L1 status.

## 2 Results

### 2.1 PD-L1-mediated IFNγ inhibition limits PD-L1 primarily for mesenchymal cells

**Figure 1:**
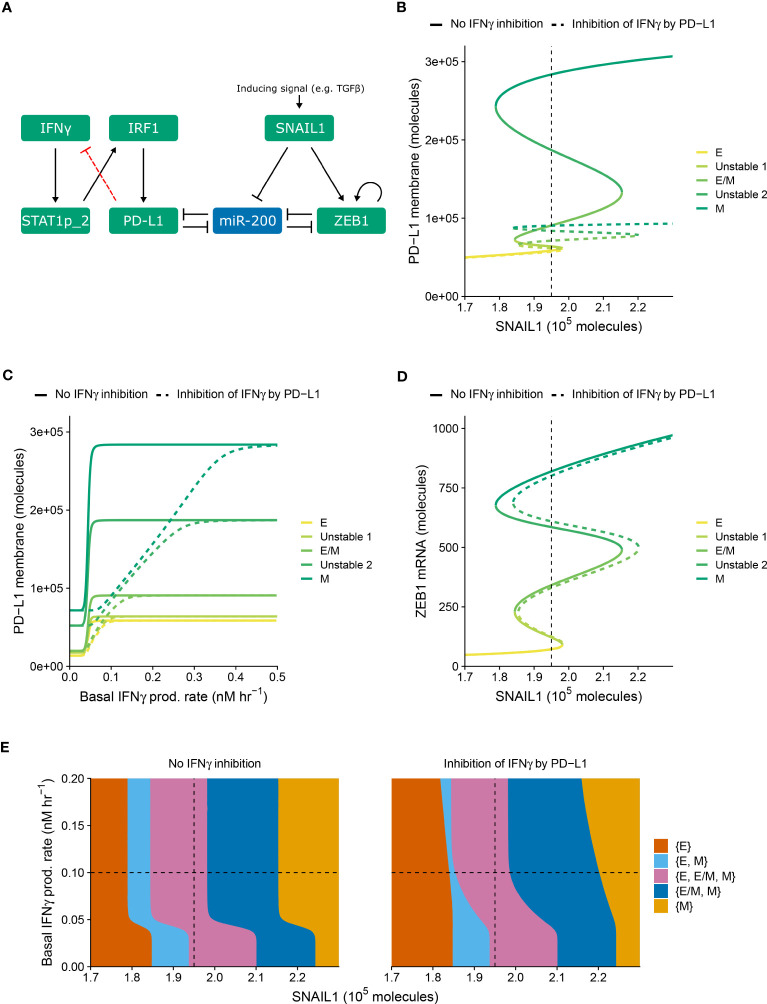
PD-L1-mediated IFNγ inhibition only quantitatively affects PD-L1 expression and EMT. **(A)** Schematic depiction of the EMT–PD-L1 regulatory network (black, solid arrows) extended with negative feedback of PD-L1 on IFNγ (red, dashed arrow). **(B–D)** Bifurcation **(B, D)** and continuation **(C)** diagrams illustrating how, in the absence (solid lines) and presence (dashed lines) of PD-L1-mediated IFNγ inhibition, the steady-state expression of PD-L1 on the membrane **(B)** and ZEB1 mRNA **(D)** depend on SNAIL1, considering a fixed basal IFNγ production rate of 0.1 nM h^−1^, and the steady-state expression of PD-L1 on the membrane depends on the basal IFNγ production rate, considering a fixed SNAIL1 level of 1.95 × 10^5^ molecules **(C)**. Colors represent the different stable equilibria (representing E, E/M, and M phenotypes) and unstable equilibria (indicated in legend). **(E)** Phase diagram showing how the presence of stable equilibria (colored regions, indicated in legend) depends on the basal IFNγ production rate and SNAIL1 in the absence (left) and presence (right) of PD-L1-mediated IFNγ inhibition. Vertical dashed lines in **(B, D, E)** show the SNAIL1 level used in **(C)**, while horizontal dashed lines in **(E)** show the basal IFNγ production rate used in **(B, D)**.

Within our previously modeled PD-L1–EMT network (**Figure 1A**, black, solid arrows), we did not consider the influence of immunosuppression. One way through which such suppression is expected to take place is the inhibition of IFNγ production following the interaction of tumor-expressed PD-L1 with T cell-expressed PD-1 (20). To study how this negative feedback of PD-L1 on IFNγ production affects the relationship between EMT and IFNγ-induced PD-L1 expression, we extended the model of Burger et al. (19) with this regulation (**Figure 1A**, red, dashed arrow).

We examined the behavior of the modified network (i.e., with PD-L1-mediated IFNγ inhibition) for various levels of SNAIL1 (considered to be activated via, e.g., TGFβ) and baseline IFNγ production rates (**Figure 1**). The model with inhibition displays similar tristability in PD-L1 expression on the cell membrane as the model without inhibition (**Figure 1B**), resulting from several saddle-node bifurcations. In both models, mesenchymal cells have the highest PD-L1 level and epithelial cells the lowest. Notably, the negative feedback loop does not cause additional bifurcation points, hence the qualitative behavior of the two models is the same. However, the feedback does decrease PD-L1 expression for all EMT phenotypes, thereby reducing the absolute and relative differences in PD-L1 expression between phenotypes. The inhibition affects the equilibrium PD-L1 level for all phenotypes when the IFNγ production rate is low, but only the mesenchymal phenotype for intermediate IFNγ production rates (**Figure 1C**). At high IFNγ production rates, the feedback has no effect on PD-L1 expression for any phenotype because the IFNγ level is still sufficiently high to closely approach the maximal transcription rate of PD-L1.

We subsequently investigated the impact of PD-L1-mediated IFNγ inhibition on ZEB1 expression and EMT phenotype stability. The inhibition causes a rightward shift of the upper part of the bifurcation diagram of ZEB1 as dependent on SNAIL1 input signal (**Figure 1D**), because a reduced PD-L1 expression leads to an increased amount of miR-200, in turn affecting EMT. To further characterize this effect, we created a phase diagram showing how the stability of EMT phenotypes depends on SNAIL1 levels and baseline IFNγ production rates (**Figure 1E**). Compared to the model without IFNγ inhibition, in the presence of such inhibition the IFNγ-induced leftward shift occurs for higher IFNγ production rates and is no longer parallel for the different bifurcation points. These bifurcation point shifts remain similar upon adjustment of the model parameters implementing the negative feedback, i.e., a sensitivity analysis (**Figure S1**, left panels). In conclusion, our model predicts that negative feedback of PD-L1 on IFNγ has a quantitative, but not qualitative, effect on the relationship between EMT and PD-L1 expression.

### 2.2 TGFβ-mediated IFNγ inhibition causes PD-L1 expression to correlate negatively with TGFβ within EMT phenotypes

**Figure 2:**
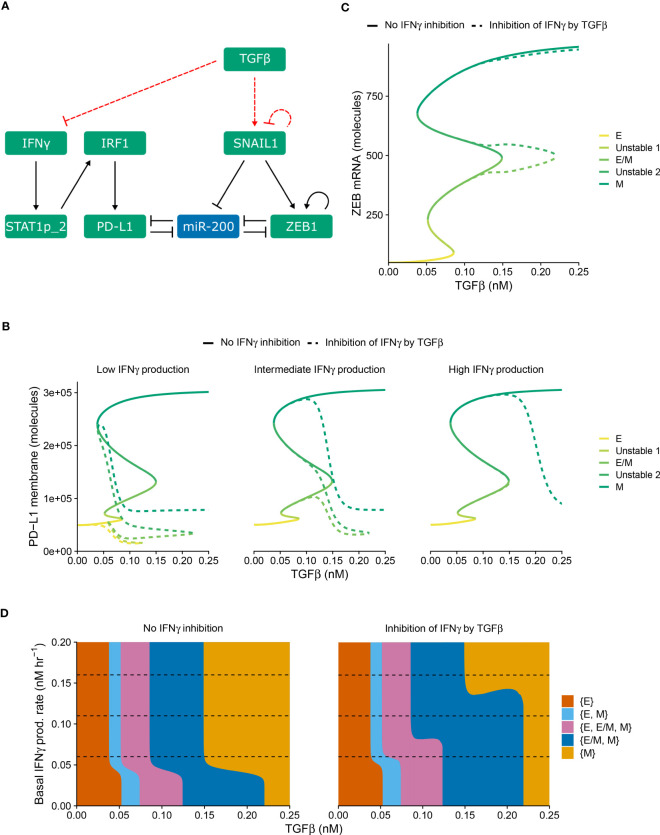
TGFβ-mediated IFNγ inhibition causes PD-L1 expression to correlate negatively with TGFβ within each EMT phenotype. **(A)** Schematic depiction of the EMT–PD-L1 regulatory network (black, solid arrows) extended with TGFβ-mediated IFNγ inhibition and SNAIL1 stimulation (red, dashed arrows). **(B, C)** Bifurcation diagrams illustrating how, in the absence (solid lines) and presence (dashed lines) of TGFβ-mediated IFNγ inhibition, the steady-state expression of PD-L1 on the membrane **(B)** and ZEB1 mRNA **(C)** depend on TGFβ, considering fixed basal IFNγ production rates of 0.06 nM h^−1^ (**B**, left), 0.11 nM h^−1^ (**B**, middle, and **C**), and 0.16 nM h^−1^ (**B**, right). Colors represent the different stable equilibria (representing E, E/M, and M phenotypes) and unstable equilibria (indicated in legend). **(D)** Phase diagram showing how the presence of stable equilibria (colored regions, indicated in legend) depends on the basal IFNγ production rate and TGFβ concentration in the absence (left) and presence (right) of TGFβ-mediated IFNγ inhibition. Horizontal dashed lines in **(D)** show the basal IFNγ production rates used in **(B, C)**.

Apart from PD-L1-mediated IFNγ inhibition leading to immunosuppression, such suppression can also be invoked by TGFβ. In order to separately study the impact of this alternative inhibition on the crosstalk between EMT and IFNγ-induced PD-L1 expression, we explicitly described TGFβ in our model as a driver of SNAIL1 expression (**Figure 2A**). Moreover, we extended this model with the inhibition of IFNγ production by TGFβ, in a similar manner as for PD-L1-mediated IFNγ production.

Using this modified model (i.e., with TGFβ-mediated IFNγ inhibition), we studied how the system responds to different levels of TGFβ and baseline IFNγ production rates (**Figure 2**). As was the case for PD-L1-mediated IFNγ inhibition, the model extension with TGFβ-mediated IFNγ inhibition does not affect the tristability of PD-L1 expression on the membrane (**Figure 2B**). However, TGFβ-mediated IFNγ inhibition leads to a complicated relation between PD-L1 expression and TGFβ. Specifically, PD-L1 levels tend to correlate negatively with TGFβ within each EMT phenotype, especially for low IFNγ production rates. Across EMT phenotypes, there is still a primarily positive correlation between TGFβ and PD-L1 expression

Next, we investigated the influence of TGFβ-mediated IFNγ inhibition on ZEB1 and the stability of EMT phenotypes. In the bifurcation diagram of ZEB1, as dependent on the TGFβ concentration (**Figure 2C**), it causes a rightward shift of the bifurcation point separating the {E/M, M} and {M} states compared with the model without inhibition. Consequently, the total range of TGFβ for which the hybrid E/M phenotype can (co-)exist is strongly increased. This is reminiscent of the influence of other proteins such as OVOL on the core EMT regulatory network (23, 24), although contrary to OVOL expression, TGFβ-mediated IFNγ inhibition does not lead to a range in which the hybrid E/M phenotype is the only possible phenotype. The increase occurs for a range of IFNγ production rates, as visualized in a phase diagram depicting the various stability regimes (**Figure 2D**). Interestingly, upon increasing the IFNγ production rate, the same bifurcation point undergoes a leftward shift, leading to a part of the curve gradually splitting off and eventually disappearing (**Figure S2**). This phenomenon also occurs for the bifurcation point separating the {E, E/M, M} and {E/M, M} states (**Figure S2**). Nevertheless, this only occurs for very limited ranges of IFNγ production rates. Importantly, also this model extension exhibits good robustness with respect to changes in inhibition-related parameter values (**Figure S1**, right panels). Moreover, when we combined both PD-L1- and TGFβ-mediated IFNγ inhibition, the effects observed for the separate inhibition mechanisms were retained (**Figure S3**). In summary, TGFβ-mediated IFNγ inhibition mainly results in a negative correlation between TGFβ and PD-L1 expression within EMT phenotypes, yet a positive correlation across phenotypes.

### 2.3 IFNγ-induced partial EMT of a tumor cell subset can provide limited protection to bystander tumor cells

In practice, the outcome of the crosstalk between EMT and IFNγ-induced PD-L1 expression is likely to also depend on the (an)isotropy of the TME with regard to the involved cytokines IFNγ and TGFβ. Therefore, we embedded our models describing IFNγ inhibition by either PD-L1 or TGFβ, or without such IFNγ inhibition, in multi-scale spatial simulations using the cellular Potts model (CPM) (25, 26). These 2D simulations comprise tumor cells, IFNγ-secreting CD8^+^ T cells, and a partial differential equation (PDE) layer describing the spatiotemporal spreading of IFNγ. The production and cellular uptake rates of IFNγ were derived from the literature (see Methods for details). Our simulations additionally include a static TGFβ field that is either uniform or has a gradient with the highest concentrations at the tumor edge. The latter mimics the accumulation of TGFβ at the invasive front which has been experimentally observed (27, 28).

Discussion is ongoing concerning how far CD8^+^ T cell-derived IFNγ can spread within the TME. Specifically, mathematical simulations predict cytokine gradients in dense, cytokine-consuming environments to range between one and a few cell diameters (29). However, these predictions are contradicted by experimental findings showing that IFNγ produced by activated CD8^+^ T cells diffuses substantially from the site of tumor cell-T cell interaction (30, 31). Since both extremes are likely relevant and can depend on tumor-secreted factors such as galectins (32), we investigated two extreme spreading scenarios by modifying the rate of cellular uptake of IFNγ. For these short- and long-range spreading scenarios, the IFNγ concentration in molecules cell^−1^ decreases by a factor of 2.7 within one and six cell layers, respectively.

**Figure 3:**
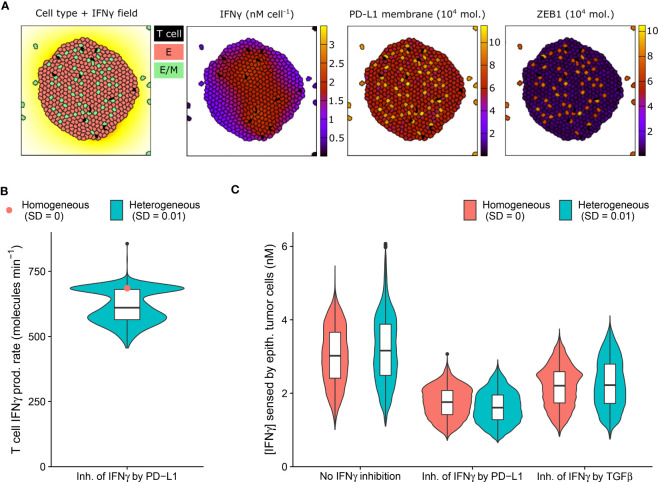
An IFNγ-induced hybrid tumor subset can provide limited protection to bystander epithelial tumor cells. **(A)** Still images of a CPM simulation of IFNγ-secreting T cells within a tumor with long-range IFNγ spreading, intratumoral heterogeneity, and PD-L1-mediated IFNγ inhibition. Left color scheme: lattice sites are colored according to IFNγ level; T cells are black, and epithelial (E) and hybrid (E/M) tumor cells are red and green, respectively. Other color schemes: T cells are black, and tumor cells are colored according to IFNγ (middle-left), PD-L1 (middle-right), and ZEB1 (right) levels. Elapsed simulation time is 2410 minutes. **(B, C)** Violin and box plots showing the IFNγ production rate of T cells **(B)** and the IFNγ concentration sensed by epithelial tumor cells **(C)**. In **(B)**, results are shown for a tumor with negative feedback of PD-L1 on IFNγ, and in **(C)** for tumors without IFNγ inhibition (left), inhibition of IFNγ by PD-L1 (middle) or by TGFβ (right). Colors denote heterogeneous (blue) or homogeneous tumors (red; only median is shown in **(B)**). Plots are based on data 2100-2410 minutes after initialization and 5 simulations per condition.

We first employed our multi-scale models to study a long-range IFNγ spreading scenario within a T cell-infiltrated tumor embedded in a uniform TGFβ field (**Figure 3A** and **Video S1**). We considered tumor cells to be either homogeneous or heterogeneous with regard to their model parameter values (see Methods), with the latter scenario likely being the most realistic for human cancers. We simulated limited heterogeneity so that no epithelial tumor cells spontaneously underwent EMT in the absence of IFNγ. Under this condition, cells also did not undergo a complete transition to a mesenchymal state in the presence of IFNγ.

IFNγ has a dual role in cancer immunity (reviewed in 33) and is implicated in tumor immune surveillance through the induction of tumor cell cycle arrest, senescence, and death. The presence of intratumoral heterogeneity makes it plausible that a subset of tumor cells is resistant to the antitumorigenic effects of IFNγ, yet is sensitive to other IFNγ-driven responses, including partial or full EMT. Because these transitions could in turn affect PD-L1 expression, inhibiting further IFNγ production, bystander tumor cells might indirectly be protected by EMT of a tumor subpopulation. We therefore investigated this potential impact of EMT triggered in a tumor subpopulation on bystander tumor cells.

As anticipated, our model predicts the entire tumor to be exposed to IFNγ due to the substantial IFNγ spreading (**Figure 3A**). Notably, the tumor cell subset that converts to an intermediate E/M state in response to IFNγ (12%) has a higher PD-L1 expression than cells remaining epithelial. In tumors with PD-L1-mediated inhibition of IFNγ secretion by neighboring T cells, this increased PD-L1 level gives rise to a clear subset of T cells with a low IFNγ production rate (**Figure 3B**). Consequently, epithelial tumor cells have on average a 7.0% lower IFNγ exposure in heterogeneous versus homogeneous tumors with PD-L1-mediated IFNγ inhibition (**Figure 3C**). Note that this small difference in sensed IFNγ by tumor cells between the homogeneous and heterogeneous scenario does not occur for tumors without IFNγ inhibition or with TGFβ-mediated IFNγ inhibition. In the scenario without IFNγ inhibition, the epithelial subpopulation is even exposed to a slightly higher (5.6%) IFNγ concentration in heterogeneous compared to homogeneous tumors. This is because several hybrid cells escape the tumor (**Figure 3A**), thereby no longer inhibiting IFNγ production of intratumoral T cells, and causing the remaining epithelial cells to reside close to the IFNγ-rich tumor center. This implies that the true effect of E/M hybrid cells on IFNγ reduction caused by the inhibition of IFNγ by PD-L1 is in fact larger than the net 7.0%. In summary, our spatial simulations provide evidence for a potential protective effect provided by a small subpopulation of hybrid tumor cells towards the remainder of the tumor population owing to PD-L1-mediated immunosuppression.

### 2.4 Population-level responses may hide the relationship between PD-L1 expression and EMT status

**Figure 4:**
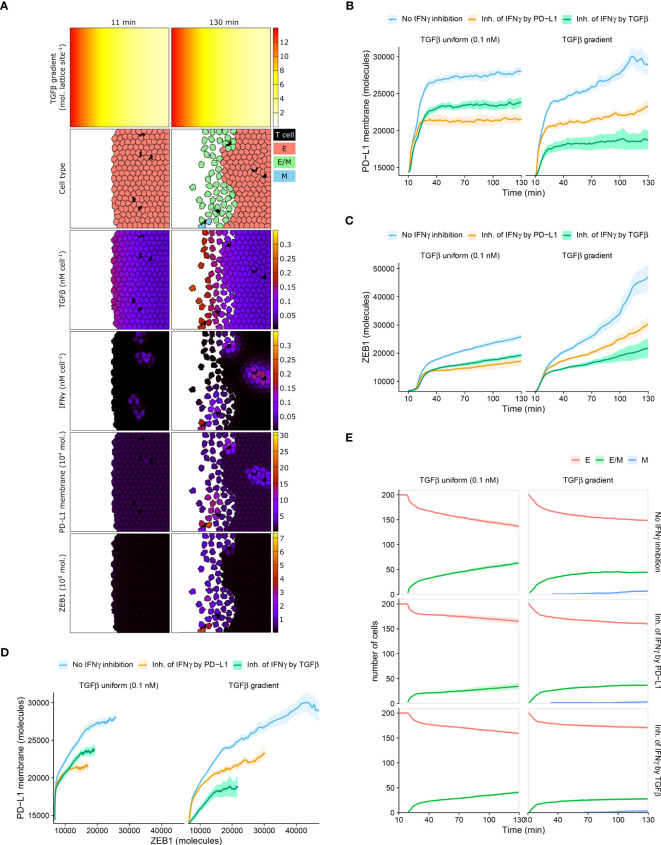
Mean PD-L1 expression need not correlate with EMT status. **(A)** Still images of a CPM simulation of IFNγ-secreting T cells at a tumor invasive front with short-range IFNγ spreading, a TGFβ gradient, and no IFNγ inhibition. Top color scheme: lattice sites are colored according to TGFβ level. Second color scheme from the top: T cells are black, and epithelial (E), hybrid (E/M), and mesenchymal (M) tumor cells are red, green, and blue, respectively. Other color schemes: T cells are black, and tumor cells are colored according to (from top to bottom) TGFβ, IFNγ, PD-L1, and ZEB1 levels. Elapsed simulation time in minutes is displayed above the stills. **(B, C)** Average (bold line) and standard error of the mean (SEM; ribbon) of PD-L1 membrane **(B)** and ZEB1 **(C)** expression of tumor cells over time. **(D)** Average (bold line) and SEM (ribbon) of PD-L1 membrane expression as a function of ZEB1 expression over time. **(E)** Average (bold line) and SEM (ribbon) of the number of tumor cells per EMT phenotype (indicated in legend) over time. Plots in **(B–E)** are based on 10 simulations per condition, and results are shown for tumors with a uniform TGFβ field (left panels) or a TGFβ gradient (right panels). The absence or mode of IFNγ inhibition is indicated in the legend.

In all investigated ODE models with or without immunosuppression, we found a clear relation between EMT and PD-L1 status, predicting PD-L1 to be lowest for epithelial cells, intermediate for hybrid E/M cells, and highest for mesenchymal cells. However, it is unclear whether this relation can be uncovered in experimental data when studying tumor cells at population level. Therefore, we investigated the relation between EMT status, ZEB1, and PD-L1 within spatial simulations implementing scenarios with short-range IFNγ spreading at the invasive front of a tumor. Note that we utilized scenarios without intratumoral heterogeneity in order to prevent this source of heterogeneity from detecting relationships between markers. Because TGFβ accumulation may occur at the invasive front in carcinomas (27, 28), we simulated tumors with either a homogeneous TGFβ field or a TGFβ gradient (**Figure 4A** and **Videos S2**, **S3**), in the absence or presence of IFNγ inhibition (either by PD-L1 or by TGFβ).

Within tumors with homogeneously distributed TGFβ or with a TGFβ gradient, the overall relationship between PD-L1 membrane and ZEB1 expression is as expected, with a higher PD-L1 expression being accompanied by a higher ZEB1 expression (**Figures 4B–D**). For instance, for tumors with a TGFβ gradient, those without IFNγ inhibition have both the highest PD-L1 and ZEB1 levels. However, between these two TGFβ tumor types, the relationship between PD-L1 and ZEB1 expression is not as straightforward. Specifically, when there is no IFNγ inhibition or PD-L1-mediated IFNγ inhibition, tumors obtain a similar level of PD-L1 expression regardless of the shape of the TGFβ field (**Figure 4B**; blue and orange), whereas tumors with a TGFβ gradient reach a much higher ZEB1 expression (**Figure 4C**; blue and orange). Moreover, in the case of IFNγ inhibition by TGFβ, tumors with a TGFβ gradient obtain a considerably lower PD-L1 (**Figure 4B**; green) but a similar ZEB1 level compared to those with a uniform TGFβ field (**Figure 4C**; green).

We subsequently examined the temporal relationship between PD-L1 membrane expression and EMT status on a single-cell level. For all tumors that are isotropic with regard to TGFβ, our models predict that the number of hybrid cells continues to increase over time (**Figure 4E**). This coincides with an increase in ZEB1 (**Figure 4C**), yet PD-L1 levels approximately reach a steady state (**Figure 4B**). This also applies to tumors with a TGFβ gradient and IFNγ inhibition by TGFβ (**Figures 4B, C, E**), although in that case the number of hybrid cells reaches a steady state. There is a minor continued increase in the number of fully mesenchymal cells in this setting (**Figure 4E**). Only in tumors with a TGFβ gradient and no immunosuppression or PD-L1-mediated IFNγ inhibition, PD-L1 expression continues to increase over time (**Figure 4B**). To conclude, an increase in the number of hybrid E/M or mesenchymal cells coincides with an increase in EMT marker ZEB1 in all studied scenarios, yet PD-L1 expression does not always keep increasing along with ZEB1. For individual tumor cells, however, we do observe the expected positive correlation between PD-L1 and ZEB1 expression in each scenario (**Figure S4**). This relation is most evident at high IFNγ levels (i.e., the top edge in each panel) in tumors with a TGFβ gradient. This implies that studying tumors at a population level may conceal the relationship between PD-L1 membrane expression and EMT status.

## 3 Discussion and conclusion

In the current study, we created mathematical and spatial models of the crosstalk between EMT and IFNγ-induced PD-L1 expression and showed that immunosuppression and heterogeneity across tumor cells and space lead to a highly complex relationship between EMT status and PD-L1 expression in cancer. Adding immunosuppression in the form of a negative feedback loop from PD-L1 on IFNγ affects this relationship only quantitatively, diminishing the differences in PD-L1 levels between the EMT phenotypes. The effect of immunosuppression through inhibition of IFNγ by TGFβ, on the other hand, results in a negative correlation between PD-L1 expression and TGFβ within each EMT phenotype. When combining PD-L1- and TGFβ-mediated IFNγ inhibition (through the multiplication of the two shifted Hill functions involved), the observed effects are consistent with those of each inhibition mechanism individually. Note that a different type of interaction between these inhibitions, such as synergism or antagonism (34), could potentially affect this outcome. Embedding the above model versions in spatial simulations of immune-infiltrated tumors, we demonstrated that IFNγ-induced partial EMT of a tumor cell subpopulation can provide limited protection to bystander tumor cells by limiting their exposure to IFNγ. Lastly, we showed that studying EMT status and PD-L1 expression at a population level may conceal their relationship. Our findings contribute to a more comprehensive understanding of the interaction between EMT and the immune response, which is essential for developing novel diagnostic and therapeutic options for cancer patients.

An interesting prediction from our models is that even though IFNγ-induced EMT gives rise to a continuous increase in average ZEB1 expression over time (**Figure 4C**), average PD-L1 expression may reach a steady state (**Figure 4B**). A potential underlying reason is that local fluctuations in IFNγ cause fluctuating PD-L1 levels that may conceal the relation between PD-L1 and ZEB1 expression (**Figure S4**). In addition, the EMT-induced upregulation of PD-L1 is relatively small compared to the initial IFNγ-induced PD-L1 upregulation. Moreover, note that our models (including the model on which our extensions are based, i.e. Burger et al. (19)) predict hybrid E/M cells to have only slightly increased (**Figure 1B**) or even lower (**Figure 2B**) PD-L1 expression compared to epithelial cells, especially in the absence of IFNγ. This is contradicted by a recent mathematical model presented by Sahoo et al. (35), which predicts an almost equal (high) level of PD-L1 for the hybrid and mesenchymal phenotypes. The model-predicted difference in PD-L1 expression between the hybrid E/M and epithelial states suggests that it is necessary to perform temporal experiments at a single-cell level to accurately capture the relationship between PD-L1 expression and EMT status (similar to **Figure S4**). Thus, future research should further characterize this difference, including its context and cell-line specificity.

The complexity of the relationship between PD-L1 expression and EMT status, and the influence of immunosuppression and spatial distribution of cytokines IFNγ and TGFβ, have relevant diagnostic implications. Both PD-L1 and EMT scores have been proposed as biomarkers for selecting patients responding to PD-1/PD-L1 blockade therapy (36, 37). However, the numerous mechanisms and factors affecting the expression of PD-L1 and EMT regulators, such as ZEB1, complicate their use as selective biomarkers (6, 38, 39). Regarding PD-L1, our model indeed predicts that a low expression may be attributed to a lack of an active immune response (initial PD-L1 level in **Figure 4B**). Alternatively, the PD-L1 level could have been high initially, suppressing the immune response and consequently decreasing the expression of PD-L1. Therefore, using PD-L1 as a predictive biomarker may prevent the treatment of a subset of patients who, despite their low to moderate PD-L1 expression, have a high probability of responding. For ZEB1 as a biomarker, a major difficulty lies in the fact that its absolute expression may depend on the shape of the TGFβ field (**Figure 4C**), as our simulations predict. Moreover, since diverse signaling pathways regulate ZEB1 activity (40), a ZEB1*^high^* tumor status is not necessarily associated with an ongoing immune response.

Furthermore, our findings support the hypothesis that T cell suppression by a hybrid E/M subpopulation in tumors with considerable IFNγ spreading may contribute to collective immunoevasion by decreasing the overall IFNγ level, albeit only slightly (**Figure 3C**). Several processes may play a role in this limited protection provided by hybrid E/M cells to other tumor cells in our simulations. First, the small effect size may partly be attributed to the aforementioned minor difference in PD-L1 expression between hybrid E/M and epithelial cells. Second, in our simulations, a substantial number of hybrid cells escape the tumor on account of their increased motility (**Figure 3A**). Note that this is in contrast with experimental observations and mathematical modeling predictions in breast carcinoma where hybrid cancer stem cells (CSCs) were found to typically reside in the tumor interior (41, 42). This distribution originated from differential EMT-inducing signals in the interior and outer regions of the tumor. Nevertheless, these findings do not exclude the possibility that hybrid (or fully mesenchymal cells) escape the tumor, as this was not specifically investigated. For example, the mathematical model of Bocci et al. (42) did not consider migration of hybrid or mesenchymal CSCs. Third, in our models we consider the IFNγ production by T cells to increase instantly upon detaching from a hybrid tumor cell. In reality, the slightly increased PD-L1 level of hybrid cells compared to epithelial cells may contribute to a sustained state of T cell exhaustion (20), resulting in long-term impaired IFNγ secretion. For these reasons, the protective effect of the hybrid tumor subset over the remainder of the tumor population may be larger than predicted here. Even if this is not the case in reality, only a minor IFNγ reduction may already be highly relevant, e.g., if it lowers the IFNγ level beyond a certain efficacy threshold of the cytopathic and cytostatic effects of IFNγ (33). If so, therapeutically targeting the hybrid subpopulation may increase the overall IFNγ concentration beyond said threshold, enhancing, e.g., the IFNγ-mediated killing of bystander epithelial tumor cells. In the future, it would therefore be useful to expand our models with the dynamics of tumor growth and T cell-mediated killing, to evaluate the importance of the predicted decrease in IFNγ. As an example of a similar approach, Benchaib et al. (43) describe tumor growth dynamics and IFNγ-induced dormancy in their mathematical model of the interaction between cancer and immune cells in the lymph node. Their simulations predict three possible outcomes that coincide with the main phases of the immunoediting process, namely tumor elimination, equilibrium, and evasion.

In our multi-scale spatial simulations, we make two more assumptions regarding T cells that would likely affect our model predictions quantitatively. First, we consider the ratio of T cells to tumor cells to be 1:40. Although this ratio represents a realistic scenario, lower ratios have been observed in some tumors, for example in glioblastoma (44). Naturally, in such tumors with very limited T cell infiltration (immunologically cold tumors), the effects predicted by our models will be less pronounced. Second, we consider T cells not to consume IFNγ. However, given that IFNγ has been shown to increase the abundance of the T cell population (45) as well as their migration and cytotoxicity (46), T cells likely take up IFNγ to a certain extent. Still, given the low T cell:tumor cell ratio, we expect that this additional consumption has only a minor effect on intratumoral IFNγ concentrations. Moreover, to our knowledge, there is no evidence indicating that T cells preferentially consume large quantities of IFNγ relative to tumor cells.

We propose that one promising therapeutic strategy for combating not only tumor immunoevasion but also cancer metastasis involves interfering with the pathways that control the interplay between EMT and PD-L1. Increasing efforts already focus on searching for opportunities to therapeutically interfere with EMT in cancer (reviewed in 47). Potential therapeutic candidates include upstream signaling pathways, such as the TGFβ signaling pathway, and molecular drivers of EMT. Blocking TGFβ signaling may also hinder its T cell-suppressive effects and is therefore an especially interesting approach. Nevertheless, our model-based analysis suggests that IFNγ is a more prominent driver of PD-L1 expression than EMT-driven PD-L1 expression *via* miR-200, which is consistent with our recent bioinformatic analysis of cancer patient data from the Cancer Genome Atlas (39). As such, we expect combination therapies of agents targeting EMT and the PD-1–PD-L1 interaction to be most effective for enhancing the antitumor immune response. Consistent with this, co-administration of TGFβ-blocking and anti-PD-L1 antibodies provoked antitumor immunity and tumor regression in metastatic urothelial cancer by facilitating T cell infiltration (48). We conclude that there is ample potential for therapeutic exploitation of the EMT–PD-L1 axis.

Our multi-scale models have three important limitations. A first limitation is that we markedly accelerated the EMT and PD-L1 regulatory network dynamics relative to their true cellular and spatial dynamics to reduce computation time. As a consequence, PD-L1 expression in our simulations was established on a time scale of seconds instead of hours, and a full EMT transition required minutes instead of days (cf. Figures 1D–F in 19). For the long-range IFNγ spreading scenario, this merely implies that in practice more time is needed for a subpopulation of hybrid cells to emerge and suppress the immune response. In actual tumors with short-range IFNγ spreading, however, the brief T cell-tumor cell interactions in our simulations might be insufficient to induce PD-L1 expression, let alone an EMT. Still, CD8^+^ T cells normally form conjugates with antigen-expressing tumor cells that can last minutes to hours (49), presumably exposing tumor cells to IFNγ for a sufficient period to induce PD-L1 expression and consequently trigger EMT.

A second limitation of our simulations is that we modeled the difference in motility between the EMT phenotypes only based on cell surface interactions, and we did not differentiate between the migratory behavior of cells in a partial EMT or mesenchymal state. Future efforts should focus on the implementation of a more sophisticated cancer invasion model, such as the cellular Potts-based model recently presented by Pramanik et al. (50), to better characterize how different modes of cell migration contribute to cancer metastasis as a consequence of EMT–PD-L1 crosstalk.

Lastly, a third limitation of our work is that we considered CD8^+^ T cells to be the only source of IFNγ in our models, even though it is well established that other immune cells in the TME can also secrete this cytokine. Examples include CD4^+^ T cells, natural killer (NK) cells, and NK T cells (51). A recent study even found the production of IFNγ by CD4^+^ chimeric antigen receptor (CAR) T cells to be considerably higher than that of CD8^+^ CAR T cells in a model of B-cell malignancy (52). Since these additional cellular components could potentially affect how our simulations replicate tumor biology, it would be worth including them (and the effects of additionally produced IFNγ) in future model versions. This also applies to the cellular sources of TGFβ, which include tumor cells, regulatory T cells, fibroblasts, and macrophages (21). We currently described this cytokine with a static field (either uniformly distributed or with a gradient) but it could instead be modeled dynamically. Note that such an effort would benefit from additional experiments to obtain reliable production and cellular uptake rates.

In conclusion, we extended an existing mathematical model and embedded it in multi-scale spatial simulations to describe the effects of immunosuppression and spatial heterogeneity on the crosstalk between EMT and IFNγ-induced PD-L1 expression. Our analysis demonstrates that the relation between PD-L1 expression and EMT status is highly complex, and depends on the forms of immunosuppression established by the tumor as well as on spatial heterogeneity concerning cytokines influencing these pathways. Experimental validation of the hypotheses presented here based on temporal, single-cell measurements will be required to shed further light on the relationship between PD-L1 expression and EMT status. Ultimately, these insights may contribute to the development of novel therapeutic strategies for effectively combating metastatic dissemination as well as immunoevasion.

## 4 Materials and methods

### 4.1 ODE models

#### 4.1.1 IFNγ–PD-L1–EMT model

The IFNγ–PD-L1–EMT model (19) uses appropriate miRNA–mRNA dynamics from the theoretical framework by Lu et al. (53) (see **Supplementary Information**) to combine the simplified TCS model (24) with a model for IFNγ-induced PD-L1 expression, which is based on an extension of a published JAK–STAT model (54). See **Supplementary Information** for the model definition and used parameters.

#### 4.1.2 Negative feedback of PD-L1 on IFNγ

Even though the negative feedback of membrane-bound PD-L1 on the production of IFNγ is not mediated by direct transcriptional regulation, for simplicity, we used a shifted Hill function to model this regulation. The shifted Hill function for activation and inhibition of A by B is defined as


(1) $$H^{S}\left(B,\lambda_{BA}\right)=H^{-}\left(B\right)+\lambda_{BA}H^{+}\left(B\right),$$

(2) $$H^{-}\left(B\right)=\frac{1}{1+{\left(\frac{B}{B_{A}^{0}}\right)}^{n_{BA}}},$$

(3) $$H^{+}\left(B\right)=1-H^{-}\left(B\right),$$

where the weight factor $\lambda_{BA}$ represents the fold change in the production rate of A due to B , with $\lambda_{BA}\;>$ 1 for activation and $\lambda_{BA}\;<$ 1 for inhibition. The Hill coefficient $n_{BA}$ represents the cooperativity of the interaction, while the threshold $B_{A}^{0}$ is the concentration of B at which the value of $H^{-}$ equals 0.5. The IFNγ–PD-L1–EMT model uses the concentration of IFNγ (in nM) as input. Here, we model the IFNγ ( I ) concentration with the following ordinary differential equation (ODE):


(4) $$\frac{dI}{dt}=g_{I}H^{S}\left(P_{M},\lambda_{P_{M},I}\right)-k_{I}I.$$

The meaning of parameters and their utilized values are provided in **Table 1**. We chose the basal production and degradation rate of IFNγ arbitrarily and varied the former to simulate different levels of IFNγ exposure. Note that upon embedding our ODE models into multi-scale spatial simulations (see below), we utilized IFNγ production and cellular uptake rates from the literature. To our knowledge, there are no experimental data available in which both IFNγ secreted by T cells and the tumor cell membrane PD-L1 expression are measured. For simplicity, we chose the value 0.1 for $\lambda_{P_{M},I}$ to allow for a considerable inhibitory effect, and the value 2 for $n_{P_{M},I}$ . $P_{M}{\;}_{I}^{0}$ was loosely based on the half-functional rule defined in Huang et al. (55), which states that a regulatory link should have an approximately equal chance of being functional or not functional. Note that we performed a sensitivity analysis to study the impact of these parameter values on the model predictions (**Figure S1**, left panels).

**Table 1:** Parameters used for the model extensions representing the immunosuppressive effects of PD-L1 and TGFβ.

		Prod. rate *g*		Degr. rate *k*		
IFNγ	*I*	*g_I_*	0-0.5 nM h^–1^	*k_I_*	1 h^–1^

	Threshold $B_{A}^{0}$		Hill coefficient *n_BA_*		Max. fold change λ*_BA_*	
Inh. *I* by *P_M_*	$P_{M}{\;}_{I}^{0}$	6×10^4^ mol.	$n_{P_{M},I}$	2	$\lambda_{P_{M},I}$	0.1
Inh. *I* by *T*	$T_{I}^{0}$	0.1 nM	$n_{T,I}$	2	$\lambda_{{\;}_{T},I}$	0.1

The top panel shows the production and degradation rate of IFNγ; the bottom panel shows parameters for the shifted Hill functions of the interactions. The parameter values were not directly obtained from the literature but were selected in this study. The production rate of IFNγ was varied to simulate different IFNγ levels.

#### 4.1.3 TGFβ–SNAIL1 model

For the TGFβ–SNAIL1 submodel, we adapted the TGFβ–miR-200 and SNAIL1–miR-34 modules of the revised CBS model (56, see **Supplementary Information**; originally published by 57). Our key modifications are the exclusion of the autocrine TGFβ–miR-200 feedback loop and the double-negative SNAIL1–miR-34 feedback loop. Because we later implement the ODE models in multi-scale models wherein tumor cells respond to extra-cellular TGFβ, our revised submodel did not need to describe TGFβ mRNA. Instead, we consider the protein TGFβ to be produced at a constant rate and to be degraded linearly, which is effectively identical to having a fixed TGFβ concentration as input. The revised TGFβ–SNAIL1 submodel consists of the following ODEs for TGFβ (*T*), SNAIL1 mRNA (*m_S_*), and SNAIL1 protein (*S*):


(5) $$\frac{dT}{dt}=g_{T}-k_{T}T,$$

(6) $$\frac{dm_{S}}{dt}=g0_{m_{S}}+g_{m_{S}}H^{+}\left(T\right)H^{-}\left(S\right)-k_{m_{S}}m_{S},$$

(7) $$\frac{dS}{dt}=g_{S}m_{S}-k_{S}S.$$

All initial conditions (i.e., the initial concentrations of T , $m_{S}$ , and S ) are set to 0. At the beginning of a simulation, the levels of TGFβ and SNAIL1 mRNA swiftly become positive because of their baseline production rates, which in turn triggers the production of SNAIL1 protein. Parameter meanings and utilized values are provided in **Table 2**. Note that, for consistency, we use *g* and *k* to denote production and degradation rates. As with IFNγ, we use arbitrary values for the production and degradation rate of TGFβ and vary the former to simulate different TGFβ exposure levels.

**Table 2:** Variables and parameters used for the TGFβ–SNAIL1 module.

		Prod. rate *g*		Degr. rate *k*	
TGFβ protein	*T*	*g_T_*	0-0.3 nM h^−1^	*k_T_*	1 h^−1^
SNAIL1 mRNA	*m_S_*	$g0_{m_{S}}$	1500 molecules h^−1^	$k_{m_{s}}$	0.09 h^−1^
		$g_{m_{S}}$	600 molecules h^−1^		
SNAIL1 protein	*S*	$g_{{\;}_{S}}$	17 h^−1^	*k_S_*	1.66 h^−1^

	Threshold $B_{A}^{0}$		Hill coefficient *n_BA_*		
Act. *m_S_* by *T*	$J_{m_{S}0}$	0.1 nM	*n*_*nt*_	2
Inh. *m_S_* by *S*	$J_{m_{S}1}$	4.0334 × 10^6^ molecules	$n_{ns}$	1

The top panel shows variable names and production and degradation rates; the bottom panel shows parameters for the Hill functions of the interactions. Parameter values were either taken from the revised CBS model by Zhang et al. (56) or modified (shade). $g0_{m_{S}}$ is the baseline production rate of SNAIL1 mRNA. The production rate of TGFβ was varied to simulate different TGFβ levels.

To create our extended model, we connected the TGFβ–SNAIL1 submodel to the central IFNγ–PD-L1–EMT model (see **Figure 2A**). Note that we converted the output SNAIL1 concentration, which was in nM in Zhang et al. (56) into number of molecules in order to use SNAIL1 as input in the IFNγ–PD-L1–EMT model. For consistency, we converted SNAIL1 mRNA to number of molecules as well. As in Jolly et al. (24) and Burger et al. (19), we use a cell volume of 10000 µm^3^, such that 1 nM amounts to approximately 6020 molecules (6.02 × 10^23^ · 10^−9^ · 10000 × (10^−5^) ^3^). To properly convert units, we thus multiplied model parameters $g0_{m_{S}}$ , $g_{m_{S}}$ , and $J_{m_{S}1}$ with 6020. In addition, we matched the range of TGFβ within which bifurcations occur to that of the CBS model by modifying parameters $g0_{m_{S}}$ , $g_{m_{s}}$ , and $J_{m_{S}0}$ .

#### 4.1.4 Inhibition of IFNγ by TGFβ

Modeling the individual components of pathways involved in the TGFβ-mediated inhibition of IFNγ secretion is a complex task. As for PD-L1-mediated IFNγ inhibition, we also used a shifted Hill function to model this regulation in a phenomenological manner. In this case, we model the IFNγ concentration (*I*) with the following ODE:


(8) $$\frac{dI}{dt}=g_{I}H^{S}\left(T,\lambda_{T,I}\right)-k_{I}I.$$

Parameter meanings and utilized values are provided in **Table 1**. In the absence of experimental data on the relationship between extra-cellular TGFβ and T cell IFNγ release, in selecting the shifted Hill function parameter values we took into account the same considerations as for the negative feedback of PD-L1 on IFNγ. We again conducted a sensitivity analysis to examine the effects of these parameter values on our model predictions (**Figure S1**, right panels).

#### 4.1.5 Combined IFNγ inhibition model

In our combined model with two forms of IFNγ inhibition, we model the dynamics of IFNγ with the following ODE:


(9) $$\frac{dI}{dt}=g_{I}H^{S}\left(P_{M},\lambda_{P_{M},I}\right)H^{S}\left(T,\lambda_{T,I}\right)-k_{I}I.$$

Note that an interesting alternative to the utilized product term of the two individual shifted Hill functions would be a combination Hill function (34), which allows for the modeling of synergistic or antagonistic effects.

### 4.2 Multi-scale models

We embedded our ODE models with separate PD-L1- or TGFβ-mediated IFNγ inhibition in multi-scale models of T cell-infiltrated tumors using the cellular Potts model (CPM) framework (25, 26), which was previously used for simulating EMT (58) and T cell-tumor cell interactions (59–62). The CPM is a lattice-based technique wherein cells consist of a collection of lattice sites that are assigned a specific ‘spin’ value to indicate their belonging to a particular cell. The models enable cellular movement through minimization of the Hamiltonian (*H*), a global energy function defined as


(10) $$H=H_{sort}+H_{l}+H_{Act}.$$

The term $H_{sort}$ describes cell surface interactions and a cell area or volume constraint that considers deviations from a target cell area or volume. As we employed two-dimensional simulations, the term ‘area’ applies here. $H_{sort}$ is calculated with the following equation:


(11) $$H_{sort}=\sum_{\begin{array}{c} \left(i,j\right)\left(i^{\prime },j^{\prime }\right)\\ neighbors \end{array}}J\left(\tau \left(\sigma \left(i,j\right)\right),\tau \left(\sigma \left(i^{\prime },j^{\prime }\right)\right)\right)\left(1-\delta_{\sigma \left(i,j\right),\sigma \left(i^{\prime },j^{\prime }\right)}\right)+\varsigma_{a}\sum_{spin\;types\;\sigma }{\left(a\left(\sigma \right)-A_{\tau \left(\sigma \right)}\right)}^{2},$$

where (i,j) and $\left(i^{\prime },j^{\prime }\right)$ are neighboring lattice sites with respective x coordinates i and $i^{\prime }$ and y coordinates j and $j^{\prime }$ , $J\left(\tau ,\tau^{\prime }\right)$ represents the surface energy between cells of types τ and $\tau^{\prime }$ , σ represents the spin of a cell, $\delta_{\sigma ,\sigma^{\prime }}$ denotes the Kronecker delta, $\varsigma_{a}$ represents a weighting term for the cell area constraint, a(σ) is the current area of a cell, and $A_{\tau \left(\sigma \right)}$ is the target area of cells with type τ . We distinguished between epithelial ( E ), hybrid E/M ( H ), and mesenchymal ( M ) tumor cells based on ZEB1 mRNA expression ( mZ ) as calculated with the ODE model. Cells transitioned as follows: E to H : mZ ≥ 235 molecules; H to E : mZ ≤ 145 molecules; H to M : mZ ≥ 715 molecules; and M to E : mZ ≤ 370 molecules. These cut-off values correspond roughly to the average expression level during each transition as predicted by our ODE models. Cells could not directly transition from a mesenchymal to a hybrid phenotype. To mimic the ‘invasion’ of hybrid and mesenchymal tumor cells, we set their surface energies with medium ( med ) lower than those with tumor cells. Conversely, we set $J_{E,med}$ higher than $J_{E,E}$ to reflect the adhesive properties of epithelial tumor cells. To prevent the migration of T cells ( Tcell ) out of the tumor, we set $J_{Tcell,med}$ higher than their surface energies with tumor cells.

The Hamiltonian of our models additionally included the term $H_{l}$ that represents the surface area constraint of cells and is calculated with the function (63)


(12) $$H_{l}=\varsigma_{l}\sum_{\sigma }{\left(l\left(\sigma \right)-L_{\tau \left(\sigma \right)}\right)}^{2},$$

where $\varsigma_{l}$ represents the weight of the perimeter constraint, l(σ) is the actual perimeter of a cell, calculated as the number of boundary interfaces with neighboring lattice sites of a different spin, and $L_{\tau \left(\sigma \right)}$ represents the target perimeter for cells with type τ . In order to promote the emergence of roundish cells, we set $L_{\tau }$ to the ratio of the perimeter of a circle to its area ( $2\sqrt{\pi A_{\tau }}$ ), with the area corresponding to the target area of a cell of type τ (following 59). Additionally, we set $\varsigma_{l_{Tcell}}$ < $\varsigma_{l_{M}}$ < $\varsigma_{l_{H}}$ < $\varsigma_{l_{E}}$ , causing T cells to deform most easily and epithelial tumor cells to most strongly retain a roundish shape.

Lastly, the active migration of T cells was driven by the term $H_{Act}$ that describes the Act model wherein actin dynamics cause cell protrusions that in turn drive cell motility (64). $H_{Act}$ is calculated with


(13) $$H_{Act}=\frac{\varsigma_{Act}}{Max_{Act}}\left(GM_{Act}\left(u\right)-GM_{Act}\left(v\right)\right),$$

where $\varsigma_{Act}$ is a weighting term of the Act model, and $Max_{Act}$ is the maximum actin activity value, which is assigned to lattice sites that are newly incorporated by a cell. The actin activity Act of a lattice site decreases with 1 after each Monte Carlo step until it reaches 0. $GM_{Act}\left(u\right)$ and $GM_{Act}\left(v\right)$ represent the geometric mean actin activities around sites u and v , respectively. The geometric mean activity around site u is calculated with


(14) $$GM_{Act}\left(u\right)={\left(\prod_{y\epsilon V\left(u\right)}Act\left(y\right)\right)}^{1/\left|V\left(u\right)\right|},$$

where |V(u)| is the second-order Moore neighborhood of site u . This implements a positive feedback mechanism that favors updates from site u into a neighboring site v with a lower actin activity. We only applied $H_{Act}$ to T cells and employed parameters for amoeboid cells (64). The resulting average migration speed was approximately 7 µm min^−1^, which is consistent with values previously measured in TC-1, EL4, and EG7 tumors (65, 66). To prevent T cells from breaking due to actin protrusion dynamics, we employed the connectivity constraint described by Merks et al. (67). Tumor cells only moved passively *via* cell surface interactions based on $H_{sort}$ and $H_{l}$ .

The simulation space comprised a square area representing the TME within which T cells and tumor cells were restricted to move. We derived the production rate of IFNγ by T cells and its rate of cellular uptake from the literature (see **Supplementary Information**). T cells were considered to continuously produce IFNγ. Because T cells were almost always in contact with tumor cells during our simulations, this is expected to closely resemble reality in which T cells may primarily produce IFNγ during periods of cognate antigen recognition. We simulated two different extents of IFNγ spreading by modifying the cellular uptake rate of IFNγ (see **Supplementary Information**). Simulations either had a uniform TGFβ field or a TGFβ gradient (see **Supplementary Information**). To enable all tumor cells to respond to extracellular TGFβ, we included the TGFβ–SNAIL1 submodel in the ODE models without IFNγ inhibition or with PD-L1-mediated IFNγ inhibition. The space had a scale of 2 µm per lattice site and was 700 × 700 µm and 400 × 400 µm in size for long-range and short-range IFNγ spreading simulations, respectively. To mimic the typically low T cell:tumor cell ratios observed within tumors (68), we simulated T cells and tumor cells at a 1:40 ratio. In long-range and short-range IFNγ spreading simulations, T cells were initiated randomly within respectively a circular tumor comprising 480 tumor cells or the middle-outer cell layers of an invasive front comprising 200 tumor cells. T cells were frozen in motion and not secreting IFNγ for the initial 10 minutes to allow tumor cell ODE dynamics to reach a steady state.

Simulations had a temporal scale of 0.6 seconds per Monte Carlo step, and output was generated every 10-minute and 1-minute interval for long-range and short-range IFNγ spreading simulations, respectively. ODE dynamics were accelerated 1800 times relative to CPM and PDE dynamics in order to make simulations less time-consuming and thus computationally feasible. CPM simulation parameters are provided in **Table 3**. In some of our simulations, we implemented intratumoral heterogeneity (see **Supplementary Information**).

**Table 3:** Cellular Potts simulation parameters.

Parameter	Value	Description	Ref.
$J_{\sigma ,\sigma^{\prime }}$	$J_{E,E}$ = 2; $J_{E,H}$ = 5; $J_{E,M}$ = 5; $J_{H,H}$ = 14; $J_{H,M}$ = 14; $J_{M,M}$ = 14; $J_{E,Tcell}$ = 0.5; $J_{H,Tcell}$ = 0.5; $J_{M,Tcell}$ = 0.5; $J_{Tcell,Tcell}$ = 0.5; $J_{E,med}$ = 3; $J_{H,med}$ = 1; $J_{M,med}$ = 1; $J_{Tcell,med,low}$ = 2; $J_{Tcell,med,high}$ = 15	Surface energies between cell types: $J_{Tcell,med,low}$ for TGFβ gradient simulations, $J_{Tcell,med,high}$ for other simulations	–
$A_{\tau }$	$A_{tum}$ = 452 µm^2^	Target area for a cell of type τ	(30, 59)
	$A_{Tcell}$ = 140 ${\text{µm}}^{2}$		
$L_{\tau }$	2 $\sqrt{\pi A_{\tau }}$	Target perimeter for a cell of type τ	(59)
$\varsigma_{a}$	$\varsigma_{a,tum}$ = 1	Strength of cell area constraint	(59)
	$\varsigma_{a,Tcell}$ = 1		
$*\varsigma_{l}$	$\varsigma_{l,E}$ = 0.25	Strength of cell perimeter constraint	(59)
	$\varsigma_{l,H}$ = 0.2		
	$\varsigma_{l,M}$ = 0.15		
	$\varsigma_{l,Tcell}$ = 0.1		
$\varsigma_{Act}$	$\varsigma_{Act,Tcell}$ = 20	Strength of actin protrusion dynamics	(64)
$Max_{Act}$	20	Actin activity value assigned to lattice sites newly occupied by T cells	(64)
$g_{I}$	1200 molecules min^−1^	Basal production rate of IFNγ by T cells	(69)
$*k_{I}$	$k_{I,Tcell}$ = 0 min^−1^	Uptake rate of IFNγ: $k_{I,short}$ for short-range IFNγ spreading simulations, $k_{I,long}$ for long-range IFNγ spreading simulations	(68, 70)
	$k_{I,tum,short}$ = 2100 min^−1^		
	$k_{I,med,short}$ = 420 min^−1^		
	$k_{I,tum,long}$ = 0.021 min^−1^		
	$k_{I,med,long}$ = 0.0042 min^−1^		
$D_{I}$	5430 µm^2^ min^−1^	Diffusion coefficient of IFNγ	(71)

The values of starred (*) parameters were based on the cited references but slightly modified. E = epithelial tumor cell; H = hybrid tumor cell; M = mesenchymal tumor cell; tum = all tumor cells independent of EMT phenotype; Tcell = T cell; med = medium.

### 4.3 Simulation and analysis

We used COPASI (COmplex PAthway SImulator) (RRID:SCR_014260) for ODE model simulations (72). For CPM simulations, we used the Morpheus framework (RRID:SCR_014975) (73). We performed analysis in R (R Project for Statistical Computing, RRID:SCR_01905) (74) with RStudio (RStudio, RRID:SCR_000432) (75) and the tidyverse (76) packages.

## Data availability statement

Data and code to run model simulations (including COPASI and Morpheus files) and generate all figures are available at https://doi.org/10.5281/zenodo.8114632 (77), further inquiries can be directed to the corresponding author.

## Author contributions

CL, GB, and JB conceptualized and designed the study. CL performed the research; GB and JB supervised the research. CL drafted the manuscript; GB and JB critically revised the manuscript. All authors read and approved the final manuscript.
