# Supplementary material for: Tumor-mediated immunosuppression and cytokine spreading affects the relation between EMT and PD-L1 status
Source: Front Immunol. 2023 Aug 10;14:1219669. doi: 10.3389/fimmu.2023.1219669 (PMC10449452; doi:10.3389/fimmu.2023.1219669)
Supplement: Supplementary file 1 [file Presentation_1.pdf]

Supplementary Information

Supplementary Methods

Theoretical framework for microRNA-TF chimera toggle-switches

Lu *et al.* [1] define the theoretical framework for microRNA-transcription factor (TF) chimera toggle-switches. For activation and inhibition of TF A by TF B, this framework uses a shifted Hill function following Eqs. (1) to (3) in the main text. The miRNA-affected translation rate of mRNA is calculated with

$$L(\mu) = \sum_{i=0}^{n} l_i C_n^i M_n^i(\mu)\,, \tag{S1}$$

where μ is the amount of miRNA, n represents the number of binding sites, l_i is the translation rate of mRNA bound to i miRNA molecules, and C_n^i and $M_n^i(\mu)$ are calculated with

$$C_n^i = \binom{n}{i} = \frac{n!}{i!(n-i)!} \tag{S2}$$

and

$$M_n^i(\mu) = \frac{\left(\frac{\mu}{\mu_0}\right)^i}{\left(1+\frac{\mu}{\mu_0}\right)^n}\,. \tag{S3}$$

In the latter equation, μ_0 represents the equilibrium constant for the binding and unbinding of miRNA to mRNA. μ_0 is calculated with

$$\mu_0 = \frac{r_{\mu-}}{r_{\mu+}}\,, \tag{S4}$$

where $r_{\mu+}$ and $r_{\mu-}$ are respectively the binding and unbinding rate of miRNA to mRNA. The total translation of mRNA is calculated with

$$L_{\text{tot}} = L(\mu) m_0\,, \tag{S5}$$

where m_0 represents the total mRNA concentration. The miRNA-facilitated mRNA degradation rate is calculated with

$$Y_m(\mu) = \sum_{i=0}^{n} \gamma_{mi} C_n^i M_n^i(\mu)\,, \tag{S6}$$

where γ_{mi} is the individual degradation rate for an mRNA bound to i miRNA. Similarly, the miRNA-facilitated miRNA degradation rate is calculated with

$$Y_\mu(\mu) = \sum_{i=0}^{n} i\gamma_{\mu i} C_n^i M_n^i(\mu)\,, \tag{S7}$$

where $\gamma_{\mu i}$ represents the individual degradation rate for a miRNA [see Supplementary Information of 1, for derivation and more details].

IFNγ–PD-L1–EMT model

The IFNγ–PD-L1–EMT model [2] combines the simplified TCS model [3] with a model for IFNγ-induced PD-L1 expression, which is based on an extension of a published JAK–STAT model [4]. The combined model consists of the following ODEs:

$$\text{miR-200}: \quad \frac{d\mu}{dt} = g_\mu H^S(Z, \lambda_{Z,\mu}) H^S(S, \lambda_{S,\mu}) - m_Z Y_{\mu,m_Z}(\mu) - m_P Y_{\mu,m_P}(\mu) - k_\mu \mu \tag{S8}$$

$$\text{mRNA ZEB1:} \quad \frac{dm_Z}{dt} = g_{m_Z} H^S(Z, \lambda_{Z,m_Z}) H^S(S, \lambda_{S,m_Z}) - m_Z Y_m(\mu) - k_{m_Z} m_Z \tag{S9}$$

$$\text{ZEB1:} \quad \frac{dZ}{dt} = g_Z m_Z L(\mu) - k_Z Z \tag{S10}$$

$$\text{IRF1 mRNA}: \quad \frac{dm_F}{dt} = g_{m_F} H^S(STAT, \lambda_{STAT,m_F}) - k_{m_F} m_F \tag{S11}$$

$$\text{IRF1 protein}: \quad \frac{dF}{dt} = g_F m_F - k_F F \tag{S12}$$

$$\text{PD-L1 mRNA}: \quad \frac{dm_P}{dt} = g_{m_P} H^S(F, \lambda_{F,m_P}) - m_P Y_m(\mu) - k_{m_P} m_P \tag{S13}$$

$$\text{PD-L1 in ER}: \quad \frac{dP_{ER}}{dt} = g_{P_{ER}} m_P L(\mu) - k_{ER,G} P_{ER} \tag{S14}$$

$$\text{PD-L1 in Golgi}: \quad \frac{dP_G}{dt} = k_{ER,G} P_{ER} - k_{G,M} P_G \tag{S15}$$

$$\text{PD-L1 membr.}: \quad \frac{dP_M}{dt} = k_{G,M} P_G - k_{P_M} P_M \tag{S16}$$

This model uses SNAIL1 and IFNγ as inputs and includes appropriate TF-TF dynamics (H^S) and miRNA-mRNA dynamics (L and Y functions) from the theoretical framework by Lu *et al.* [1]. The parameters used in the $L(\mu), Y_m(\mu)$, and $Y_\mu(\mu)$ functions are shown in Table S1 [following 2]. Table S2 lists the variables and remaining parameters used in the model.

For (IFNγ)–JAK–STAT signaling, the IFNγ–PD-L1–EMT model uses a steady-state approximation of the JAK–STAT model by Quaiser *et al.* [4]. The relationship between IFNγ and [STAT1p_2] is described with a Gompertz function:

$$f(x) = d\, \mathrm{e}^{-\mathrm{e}^{b(x-e)}}, \tag{S17}$$

with parameter values $d = 414.56394$, $b = -51.52794$, and $e = 0.02833$. [STAT1p_2] in nM is converted to STAT1p_2 in number of molecules by multiplying with 6020 following Jolly *et al.* [3]. Table S3 shows all components in the IFNγ–PD-L1–EMT model and their units.

Cascading Bistable Switches model

The revised CBS model ([5]; [originally presented by 6]) is built on a theoretical framework for miRNA-mediated regulation of mRNA different from the one defined in Lu *et al.*

Table S1: Rates used in the $L(\mu), Y_m(\mu)$, and $Y_\mu(\mu)$ functions in the IFNγ–PD-L1–EMT model. The top panel shows rates used in the simplified TCS model for $\mu_0 = 10^4$ molecules and $n = 6$ [3]; the bottom panel shows rates used in the combined model for $\mu_0 = 10^4$ molecules and $n = 2$ [2].

n (# of miRNA binding sites)	0	1	2	3	4	5	6
l_i [h^{-1}]	1	0.6	0.3	0.1	0.05	0.05	0.05
γ_{mi} [h^{-1}]	0	0.04	0.2	1	1	1	1
$\gamma_{\mu i}$ [h^{-1}]	0	0.005	0.05	0.5	0.5	0.5	0.5

n (# of miRNA binding sites)	0	1	2
l_i [h^{-1}]	1	0.3	0.05
γ_{mi} [h^{-1}]	0	0.2	1
$\gamma_{\mu i}$ [h^{-1}]	0	0.05	0.5

Table S2: Parameters used for the IFNγ–PD-L1–EMT model [2]. The top panel shows production and degradation rates; the bottom panel shows parameters for the shifted Hill functions of the interactions. Production rates g are in molecules h^{-1}, degradation rates k in h^{-1}, and thresholds B_A^0 in molecules.

		Prod. rate g		Degr. rate k	
miR-200	μ	g_μ	2100	k_μ	0.0475
mRNA ZEB1	m_Z	g_{m_Z}	11	k_{m_Z}	0.5
ZEB1	Z	g_Z	100	k_Z	0.1
IRF1 mRNA	m_F	g_{m_F}	30	k_{m_F}	0.5
IRF1 protein	F	g_F	100	k_F	0.1
PD-L1 mRNA	m_P	g_{m_P}	30	k_{m_P}	0.5
PD-L1 in ER	P_{ER}	$g_{P_{ER}}$	100	$k_{ER,G}$	1.68
PD-L1 in Golgi Complex	P_G			$k_{G,M}$	1.8
PD-L1 on membrane	P_M			k_{P_M}	0.15

	Threshold B_A^0		Hill coefficient n_{BA}		Max. fold change λ_{BA}	
Inh. μ by Z	Z_μ^0	2.2×10^5	$n_{Z,\mu}$	3	$\lambda_{Z,\mu}$	0.1
Inh. μ by S	S_μ^0	1.8×10^5	$n_{S,\mu}$	2	$\lambda_{S,\mu}$	0.1
Act. m_Z by Z	$Z_{m_Z}^0$	2.5×10^4	n_{Z,m_Z}	2	λ_{Z,m_Z}	7.5
Act. m_Z by S	$S_{m_Z}^0$	1.8×10^5	n_{S,m_Z}	2	λ_{S,m_Z}	10
Act. m_F by $STAT$	$STAT_{m_F}^0$	2×10^6	n_{STAT,m_F}	10	λ_{STAT,m_F}	10
Act. m_P by F	$F_{m_P}^0$	10^5	n_{F,m_P}	3	λ_{F,m_P}	10

Table S3: List of regulators in the IFNγ–PD-L1–EMT model [2]. Starred (*) regulators IFNγ and SNAIL1 are the two model inputs.

Regulator	Symbol	Units
*extra-cellular IFNγ	$[IFN\gamma]$	nM
[STAT1p_2]	x_{10}	nM
STAT1p_2	$STAT$	# molecules
IRF1 mRNA	m_F	# molecules
IRF1 protein	F	# molecules
PD-L1 mRNA	m_P	# molecules
PD-L1 in Endoplasmatic Reticulum	P_{ER}	# molecules
PD-L1 in Golgi Complex	P_G	# molecules
PD-L1 on cell membrane	P_M	# molecules
miR-200	μ	# molecules
ZEB1 mRNA	m_Z	# molecules
ZEB1 protein	Z	# molecules
*SNAIL1 protein	S	# molecules

[1]. In this framework, activation and inhibition of TF A by TF B are defined by Hill functions (Eqs. (2) and (3)). General miRNA, mRNA, and protein dynamics are modeled with

$$\text{Total miRNA}: \quad \frac{d[miRNA]_t}{dt} = k_{miR} - kd_{miR}[miRNA] - kd_{R1}(1-\lambda)[R1]\,, \tag{S18}$$

$$\text{Total mRNA}: \quad \frac{d[mRNA]_t}{dt} = k_{mR} - kd_{mR}[mRNA] - kd_{R1}[R1]\,, \tag{S19}$$

$$\text{Protein}: \quad \frac{d[Protein]}{dt} = k_{s0}[mRNA] + k_{s1}[R1] - kd_{Protein}[Protein]\,, \tag{S20}$$

(S21)

where k and kd denote respectively production and degradation rates, $[miRNA]$ and $[mRNA]$ are the amount of free miRNA and mRNA, respectively, λ is the recycle ratio for an miRNA following degradation of the miRNA-mRNA complex R1, and k_{s0} and k_{s1} are the translation rates of free mRNA and the R1 complex, respectively. $R1$ is calculated with

$$R1 = K[miRNA][mRNA]\,. \tag{S22}$$

In this equation, the equilibrium constant K is calculated with

$$K = \frac{k_{on}}{k_{off}}\,, \tag{S23}$$

where k_{on} and k_{off} are the binding and unbinding rates of miRNA to mRNA, respectively. The amount of miRNA-mRNA complex with i miRNAs bound is calculated with

$$R_i = K[miRNA][R_{i-1}]\,. \tag{S24}$$

Conservation equations for miRNA and mRNA are

$$[miRNA]_t = [miRNA] + \sum_{i=0}^{n} iC_n^i R_i \tag{S25}$$

and

$$[mRNA]_t = [mRNA] + \sum_{i=0}^{n} C_n^i R_i \,, \tag{S26}$$

where n is the number of binding sites, and C_n^i is calculated with Eq. (S2) [see Supplementary Information of 5, for derivation and more details].

The TGFβ and SNAIL1–miR-34 modules of the revised CBS model consist of the following equations:

$$\begin{aligned}
\text{Total TGF}\beta\text{ mRNA}: \quad \frac{d[tgf]_t}{dt} &= k_{tgf} - kd_{tgf}[tgf] - kd_{TR}[TR] && \text{(S27)}\\
\text{Free TGF}\beta\text{ mRNA}: \quad [tgf] &= [tgf]_t - [TR] && \text{(S28)}\\
\text{TR complex}: \quad [TR] &= K_{TGF}[miR\text{-}200][tgf] && \text{(S29)}\\
\text{Autocrine TGF}\beta\text{ protein}: \quad \frac{d[TGF]}{dt} &= k_{TGF}[tgf] - kd_{TGF}[TGF] && \text{(S30)}\\
\text{Total TGF}\beta\text{ protein}: \quad [TGF]_t &= [TGF] + [TGF0] && \text{(S31)}\\
\text{Total miR-34}: \quad \frac{d[miR\text{-}34]_t}{dt} &= k0_{34} + k_{34}H^-(SNAIL1)H^-(ZEB) && \\
&\quad - kd_{34}[miR\text{-}34] - (1-\lambda_s)kd_{SR1}[SR] && \text{(S32)}\\
\text{Free miR-34}: \quad [miR\text{-}34] &= [miR\text{-}34]_t - [SR] && \text{(S33)}\\
\text{Total SNAIL1 mRNA}: \quad \frac{d[snail1]_t}{dt} &= k0_{snail} + k_{snail}H^+(TGF)H^-(SNAIL1) && \\
&\quad - kd_{snail1}[snail1] - kd_{SR}[SR] && \text{(S34)}\\
\text{Free SNAIL1 mRNA}: \quad [snail1] &= [snail1]_t - [SR] && \text{(S35)}\\
\text{SR complex}: \quad [SR] &= K_s[snail1][miR\text{-}34] && \text{(S36)}\\
\text{SNAIL1 protein}: \quad \frac{d[SNAIL1]_t}{dt} &= k_{SNAIL}[snail1] - kd_{SNAIL}[SNAIL1] && \text{(S37)}
\end{aligned}$$

In the TGFβ module, $[TGF0]$ represents the input concentration of exogenous TGFβ in µM. Table S4 lists the variables and parameters used in the TGFβ and SNAIL1–miR-34 modules.

IFNγ

Because PD-L1 expression on the tumor cell membrane should depend on the local IFNγ level, we simulated IFNγ diffusion in our multi-scale models using a PDE layer describing the IFNγ concentration. Within the Morpheus framework, secretion and degradation rates are expressed per lattice site [7]. Hence, we divided the T cell production and

Table S4: Variables and parameters used for the TGFβ and SNAIL1–miR-34 modules of the revised CBS model [5]. The top panel shows production and degradation rates; the middle panel shows parameters for the miRNA-mRNA complexes; the bottom panel shows parameters for the Hill functions of the interactions. Production rates k are in µM h^{-1}, degradation rates kd in h^{-1}, k_{on}/k_{off} constants K in µM^{-1}, and thresholds B_A^0 in µM. The starred (*) parameter was not explicitly named by Zhang *et al.* [5] or Tian *et al.* [6].

		Prod. rate k		Degr. rate kd	
TGFβ mRNA	*tgf*	k_{tgf}	0.05	kd_{tgf}	0.09
TGFβ protein	*TGF*	k_{TGF}	1.6	kd_{TGF}	1
SNAIL1 mRNA	*snail1*	$k0_{snail}$	6×10^{-4}	kd_{snail}	0.09
		k_{snail}	0.05		
SNAIL1 protein	*SNAIL1*	k_{SNAIL}	17	kd_{SNAIL}	1.66
miR-34	*miR-34*	$k0_{34}$	1.2×10^{-3}	kd_{34}	0.035
		k_{34}	0.012		

	K (k_{on}/k_{off})		Degr. rate kd		Recycle ratio λ	
TGFβ mRNA–miR-200 (TR) complex	K_{TGF}	20	kd_{TR}	0.9		
SNAIL1 mRNA–miR-34 (SR) complex	K_S	100	kd_{SR}	0.9	λ_s	0.5

	Threshold B_A^0		Hill coefficient n_{BA}	
Act. *snail1* by *TGF*	J_{snail0}	0.62	n_{nt}	2
Inh. *snail1* by *SNAIL1*	J_{snail1}	0.67	$^*n_{ns}$	1
Inh. *miR-34* by *SNAIL1*	$J1_{34}$	0.15	n_{ns}	2
Inh. *miR-34* by *ZEB*	$J2_{34}$	0.36	n_{nz}	2

tumor cell uptake rates of IFNγ (explained below) by the actual area of T cells and the target area of tumor cells, respectively. IFNγ is primarily secreted by activated lymphocytes such as $CD8^+$ and $CD4^+$ T cells, natural killer (NK) cells, and NK T cells [8]. For simplicity, we considered tumor-infiltrating $CD8^+$ T cells to be the only source of IFNγ in our model. T cells secrete IFNγ at an average rate of $1200\,\text{molecules}\,\text{min}^{-1}$ for several hours after activation [9]. Therefore, we set the baseline production of IFNγ by T cells at this rate. In simulations with PD-L1-mediated inhibition of IFNγ, the total IFNγ production rate of a T cell depended on the average PD-L1 level of its neighboring lattice sites, following Eq. (4) in the main text. In simulations with TGFβ-mediated inhibition of IFNγ, the total IFNγ production rate of a T cell depended on its local TGFβ concentration, calculated as described in the next section, following Eq. (8) in the main text. Produced IFNγ was distributed over all lattice sites covered by the generating T cell.

Upon secretion, IFNγ diffused into the TME with a diffusion coefficient of $5.43 \times 10^3\,\mu\text{m}^2\,\text{min}^{-1}$, which was previously measured *in vivo* in murine lymph node tissue [10]. As it remains unclear to what extent T cell-derived IFNγ can spread within the TME, and this likely depends on TME composition [11], we simulated two different extents of IFNγ spreading by modifying the rate of cellular uptake of IFNγ by tumor cells. This rate has previously been estimated at $2.1\,\text{min}^{-1}$ based on an *in vitro* experiment with cultured fibroblasts [12, 13]. We opted to consider differential uptake rates to describe the two spreading scenarios because the uptake rate may well be different in *in vivo* tumors compared to *in vitro* fibroblasts. We modeled the long- and short-range spreading of IFNγ with uptake rates of $2.1 \times 10^{-2}\,\text{min}^{-1}$ and $2.1 \times 10^3\,\text{min}^{-1}$, respectively. Using these rates, the IFNγ concentration in molecules cell^{-1} decreases by a factor of 2.7 within 6 and 1 cell layer(s), respectively, when simulating a single IFNγ-producing T cell surrounded by IFNγ-consuming tumor cells. Following Beck *et al.* [13], we set the rate of disappearance of IFNγ in the medium at 20% of the cellular uptake rate of tumor cells. We considered T cells not to consume IFNγ. The Morpheus framework employs a Forward-Euler diffusion solver that has a time-step limit depending on the diffusion rate [7]. To make our simulations feasible with regard to the computation time involved, we decreased the diffusion coefficient and cellular uptake rates by a factor of 100 (i.e., the earlier-mentioned values for these parameters were divided by 100). Importantly, this only influences the absolute IFNγ concentration and the time until a steady state is reached, and not the extent of IFNγ spreading.

We approximated the local IFNγ concentrations in nM cell^{-1} by dividing the total number of molecules located on the lattice sites belonging to a cell by the volume that the cell would have in three dimensions based on a diameter of $24\,\mu\text{m}$ [14] and considering tumor cells to be perfect spheres. This is the diameter of a circular tumor cell with an area that equals the tumor cell target area. The resulting IFNγ concentration was used as an input signal for the ODE model. For short-range IFNγ spreading simulations, the calculated IFNγ concentrations were lowered (by multiplying them with 0.6) to create a concentration range that led to a limited amount of phenotype-switching events roughly consistent with the low numbers of E/M and M tumor cells in *in vivo* tumors. We employed the Neumann boundary condition representing zero flux at the boundary in all

simulations. For long-range IFNγ spreading simulations, we additionally set the IFNγ disappearance rate in lattice sites along the simulation space boundary to $1\,\text{min}^{-1}$ to prevent IFNγ accumulation.

TGFβ

Simulations either had a uniform TGFβ field or a TGFβ gradient. We modeled the former by setting the TGFβ concentration in nM cell^{-1} as a fixed parameter instead of explicitly modeling a field. The TGFβ concentration was $0.07\,\text{nM}$ cell^{-1} and $0.1\,\text{nM}$ cell^{-1} for long- and short-range IFNγ spreading simulations, respectively. These parameters were taken differently in order to tune the phenotype switching rates between these two scenarios. We modeled the TGFβ gradient with a TGFβ field varying between approximately 13.7 and 2.1 molecules per lattice site from left to right in the simulation space. The local TGFβ concentration in nM cell^{-1} was calculated by dividing the sum of all TGFβ molecules located on the sites occupied by the cell by the estimated volume the cell would have had in three dimensions. This volume was calculated based on a diameter determined from either the cell's target area (for tumor cells) or the cell's actual area (for T cells), considering cells to be perfect spheres.

Intratumoral heterogeneity

We implemented intratumoral heterogeneity by randomly drawing all ODE model parameter values initially assigned to tumor cells from a Gaussian distribution with a mean of the default parameter value and a standard deviation (SD) of 1% of the default parameter value. We selected such a low SD because a greater SD triggered spontaneous EMT in a subset of tumor cells even in the absence of IFNγ. For parameters with either a positive or negative default value, drawing of parameter values from a Gaussian distribution for individual tumor cells could hypothetically lead to a negative or positive parameter value. Due to the relatively small SD, the probability of such events was only approximately $1.34 \times e^{-2174}$ for any given parameter. In these rare cases, a value of 0 was used for the tumor cell. We additionally mimicked heterogeneity in IFNγ and TGFβ cell surface receptor expression by multiplying the sensed IFNγ and TGFβ concentrations by two additional parameters with a default value of 1 and heterogeneity as described above.

References

1. Lu, M. *et al.* Tristability in cancer-associated microRNA-TF chimera toggle switch. *J. Phys. Chem. B* **117,** 13164–13174. doi:10.1021/jp403156m (2013).

2. Burger, G. A., Nesenberend, D. N., Lems, C. M., Hille, S. C. & Beltman, J. B. Bidirectional crosstalk between epithelial-mesenchymal plasticity and IFNγ-induced PD-L1 expression promotes tumour progression. *Royal Society Open Science* **9.** doi:10.1098/RSOS.220186 (11 2022).

3. Jolly, M. K. *et al.* Stability of the hybrid epithelial/mesenchymal phenotype. *Oncotarget* **7,** 27067–27084. doi:10.18632/oncotarget.8166 (2016).

4. Quaiser, T., Dittrich, A., Schaper, F. & Mönnigmann, M. A simple work flow for biologically inspired model reduction - application to early JAK-STAT signaling. *BMC Systems Biology* **5,** 30. doi:10.1186/1752-0509-5-30 (2011).

5. Zhang, J. *et al.* TGF-β-induced epithelial-to-mesenchymal transition proceeds through stepwise activation of multiple feedback loops. *Science Signaling* **7,** ra91. doi:10.1126/scisignal.2005304 (2014).

6. Tian, X. J., Zhang, H. & Xing, J. Coupled reversible and irreversible bistable switches underlying TGFβ-induced epithelial to mesenchymal transition. *Biophysical Journal* **105,** 1079–1089. doi:10.1016/j.bpj.2013.07.011 (2013).

7. Starruß, J., De Back, W., Brusch, L. & Deutsch, A. Morpheus: A user-friendly modeling environment for multiscale and multicellular systems biology. *Bioinformatics* **30,** 1331–1332. doi:10.1093/bioinformatics/btt772 (2014).

8. Burke, J. D. & Young, H. A. IFN-γ: A cytokine at the right time, is in the right place. *Seminars in Immunology* **43,** 101280. doi:https://doi.org/10.1016/j.smim.2019.05.002 (2019).

9. Han, Q. *et al.* Polyfunctional responses by human T cells result from sequential release of cytokines. *Proceedings of the National Academy of Sciences of the United States of America* **109,** 1607–1612. doi:10.1073/pnas.1117194109 (2012).

10. Ross, A. E. & Pompano, R. R. Diffusion of cytokines in live lymph node tissue using microfluidic integrated optical imaging. *Analytica Chimica Acta* **1000,** 205–213. doi:10.1016/j.aca.2017.11.048 (2018).

11. Hoekstra, M. E., Vijver, S. V. & Schumacher, T. N. Modulation of the tumor microenvironment by CD8+ T cell-derived cytokines. *Current Opinion in Immunology* **69,** 65–71. doi:10.1016/j.coi.2021.03.016 (2021).

12. Anderson, P., Yip, Y. K. & Vilcek, J. Human interferon-γ is internalized and degraded by cultured fibroblasts. *Journal of Biological Chemistry* **258,** 6497–6502. doi:10.1016/s0021-9258(18)32439-6 (1983).

13. Beck, R. J., Slagter, M. & Beltman, J. B. Contact-dependent killing by cytotoxic T lymphocytes is insufficient for EL4 tumor regression in vivo. *Cancer Research* **79,** 3406–3416. doi:10.1158/0008-5472.CAN-18-3147 (2019).

14. Hoekstra, M. E. *et al.* Long-distance modulation of bystander tumor cells by CD8+ T-cell-secreted IFN-γ. *Nature Cancer* **1,** 291–301. doi:10.1038/s43018-020-0036-4 (2020).

Figures

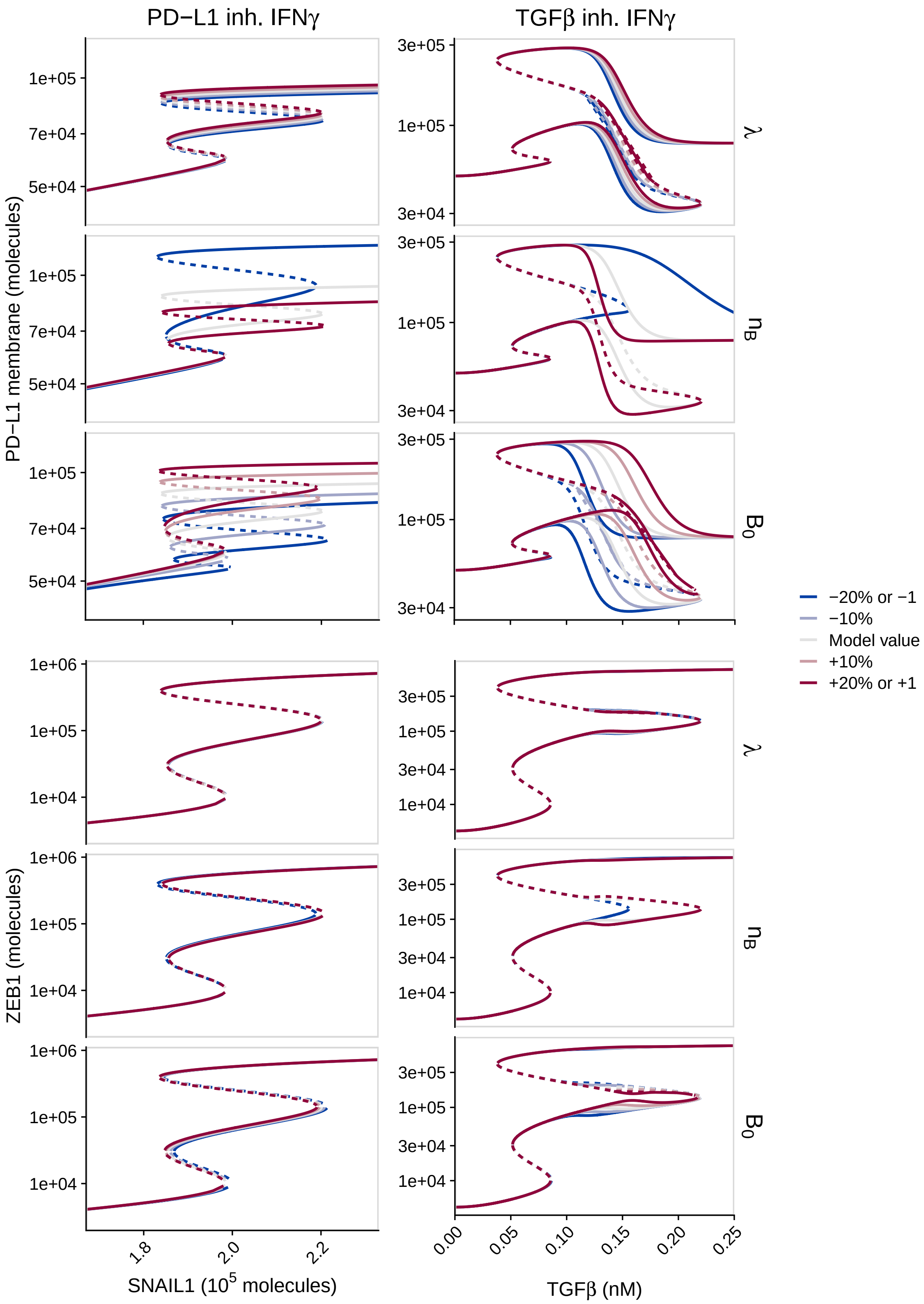

PD−L1 inh. IFNγ
TGFβ inh. IFNγ
PD−L1 membrane (molecules)
ZEB1 (molecules)
λ
nB
B0
−20% or −1
−10%
Model value
+10%
+20% or +1
1e+05
7e+04
5e+04
3e+05
3e+04
1e+06
1e+04
1.8
2.0
2.2
0.00
0.05
0.10
0.15
0.20
0.25
SNAIL1 (10^5 molecules)
TGFβ (nM)

Figure S1 (displayed on previous page)**:** Sensitivity analysis of PD-L1 on the membrane and ZEB1 protein for parameters used in the inhibition of IFNγ production by PD-L1 and TGFβ. Modifications in the bifurcation diagram are shown for PD-L1 (top) and ZEB1 (bottom) as dependent on SNAIL1 (left) or TGFβ (right) but for a fixed baseline IFNγ production rate of $0.1\,\text{nM}\,\text{h}^{-1}$ (left) or $0.11\,\text{nM}\,\text{h}^{-1}$ (right). Parameters B_A^0 and λ_{BA} for the H^S functions (see Table 1) were varied from the default model values (black) by +10/20% (red shades) and -10/20% (blue shades), while n_{BA} parameters for these functions were varied from the default model values (black) by +1 (red) and -1 (blue).

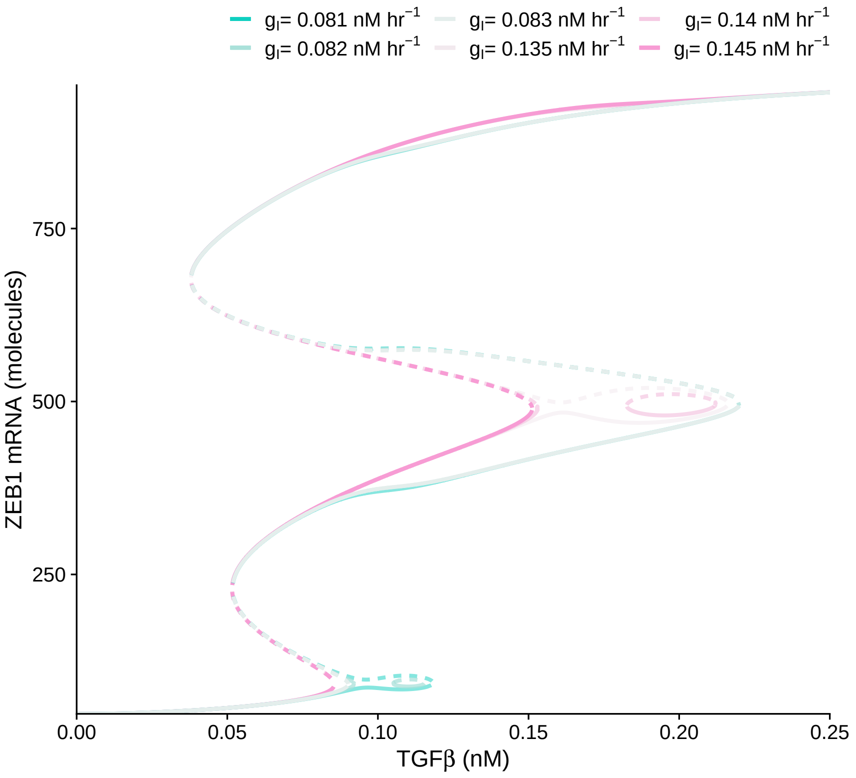

Figure S2: Bifurcation diagram illustrating how ZEB1 mRNA depends on TGFβ in our model extended with the inhibition of IFNγ by TGFβ for different basal IFNγ production rates g_I (see Eq. (8) in main text).

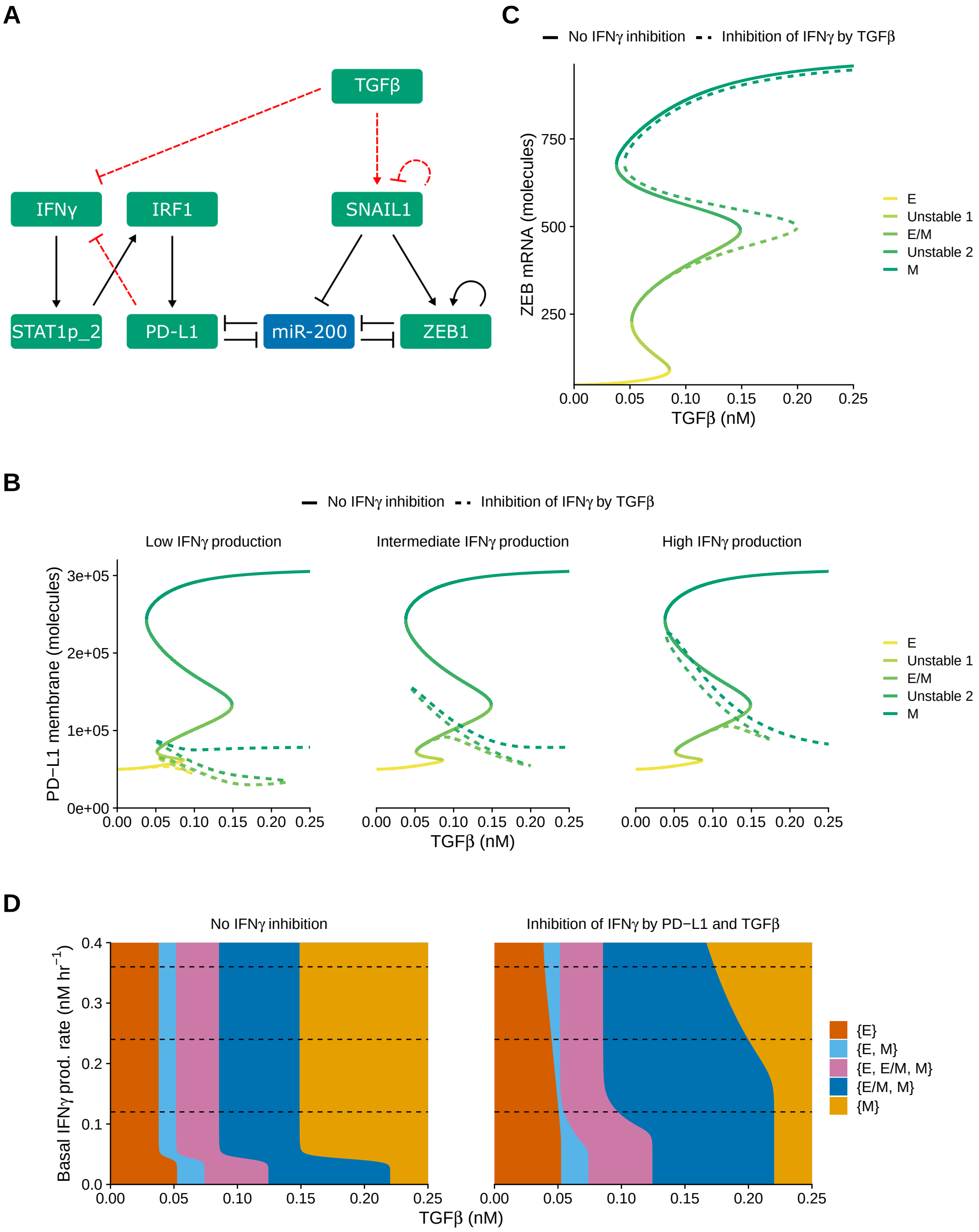

Figure S3: Model-predicted influence of combined PD-L1- and TGFβ-mediated IFNγ inhibition on PD-L1 expression and EMT. (A) Schematic depiction of the EMT–PD-L1 regulatory network (black, solid arrows) extended with the inhibition of IFNγ by PD-L1 and TGFβ and SNAIL1 stimulation (red, dashed arrows). (B)-(C) Bifurcation diagrams illustrating how, in the absence (solid lines) and presence (dashed lines) of the inhibition of PD-L1 and TGFβ on IFNγ, the steady-state expression of PD-L1 on the membrane (B) and ZEB1 mRNA (C) depend on TGFβ, considering fixed basal IFNγ production rates of $0.12\,\mathrm{nM\,h^{-1}}$ (B, left), $0.24\,\mathrm{nM\,h^{-1}}$ (B, middle, and C), and $0.36\,\mathrm{nM\,h^{-1}}$ (B, right). Colors represent the different stable equilibria (representing E, E/M, and M phenotypes) and unstable equilibria (indicated in legend). (D) Phase diagram showing how the presence of stable equilibria (colored regions, indicated in legend) depends on the basal IFNγ production rate and TGFβ in the absence (left) and presence (right) of inhibition of PD-L1 and TGFβ on IFNγ. Horizontal dashed lines in (D) show the basal IFNγ production rates used in (B) and (C).

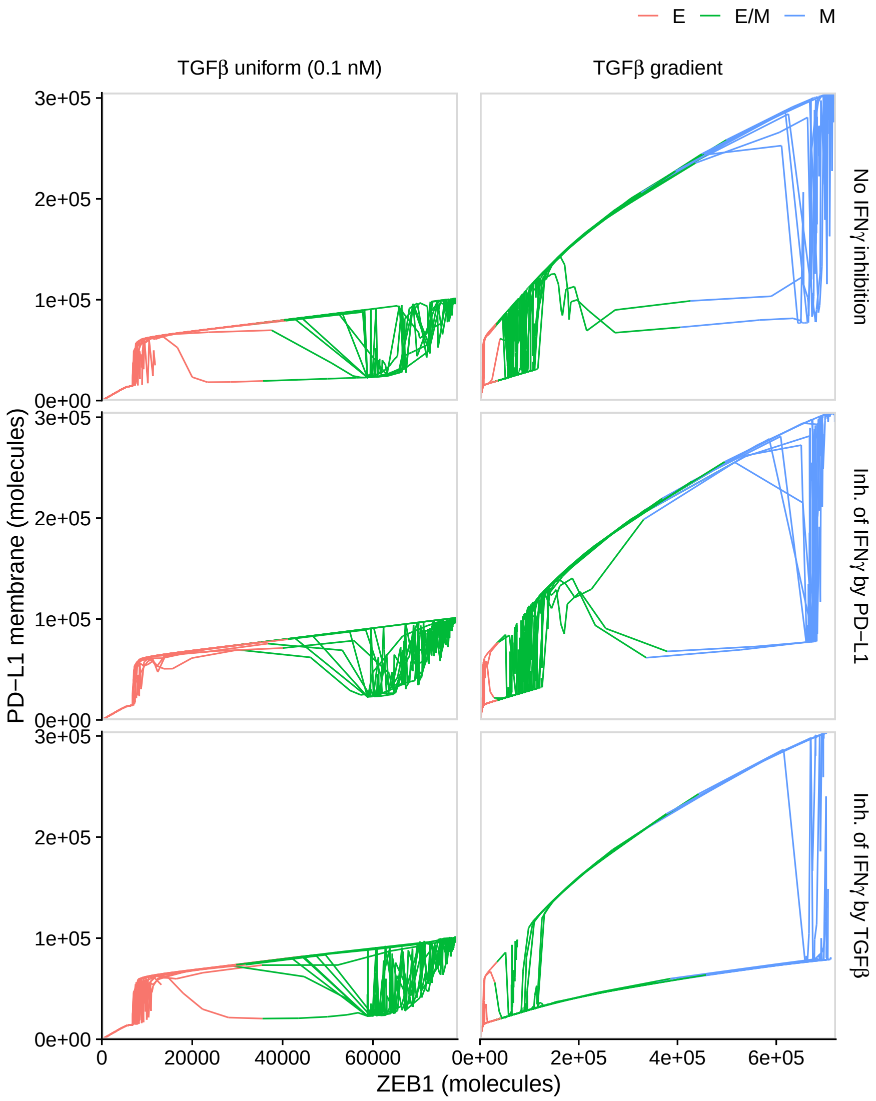

Figure S4: PD-L1 membrane expression as a function of ZEB1 expression over time for individual tumor cells. Per EMT phenotype, as achieved at the end of the simulation time, 10 randomly selected cells are shown. Results are shown for tumors with a uniform TGFβ field (left panels) or a TGFβ gradient (right panels), without IFNγ inhibition (top panels) or with inhibition of IFNγ by either PD-L1 (middle panels) or TGFβ (bottom panels).

Videos

Video S1. IFNγ-secreting T cells within a tumor with long-range IFNγ spreading, intratumoral heterogeneity, and negative feedback of PD-L1 on IFNγ. Top left color scheme: lattice sites are colored according to IFNγ level. T cells are black, and epithelial and hybrid tumor cells are red and green, respectively. Top right color scheme: lattice sites are colored according to IFNγ production rate in T cells, with tumor cells in black. Other color schemes: T cells are black, and tumor cells are colored according to TGFβ concentration (top center), IFNγ concentration (bottom left), PD-L1 (bottom center), and ZEB1 (bottom right). Elapsed simulation time in minutes is displayed in the lower right corner. Also available in higher quality at `https://youtu.be/FnCDWHjYsq4`.

Video S2. IFNγ-secreting T cells at a tumor invasive front with short-range IFNγ spreading, a uniform TGFβ field, and absence of IFNγ inhibition. Top left color scheme: lattice sites are colored according to IFNγ level. T cells are black, and epithelial and hybrid tumor cells are red and green, respectively. Top right color scheme: lattice sites are colored according to IFNγ production rate in T cells, with tumor cells in black. Other color schemes: T cells are black, and tumor cells are colored according to TGFβ concentration (top center), IFNγ concentration (bottom left), PD-L1 (bottom center), and ZEB1 (bottom right). Elapsed simulation time in minutes is displayed in the lower right corner. Also available in higher quality at `https://youtu.be/XGqelwz7luE`.

Video S3. IFNγ-secreting T cells at a tumor invasive front with short-range IFNγ spreading, a TGFβ gradient, and absence of IFNγ inhibition. Top left color scheme: lattice sites are colored according to TGFβ level. T cells are black, and epithelial, hybrid, and mesenchymal tumor cells are red, green, and blue, respectively. Top right color scheme: lattice sites are colored according to IFNγ production rate in T cells, with tumor cells in black. Other color schemes: T cells are black, and tumor cells are colored according to TGFβ concentration (top center), IFNγ concentration (bottom left), PD-L1 (bottom center), and ZEB1 (bottom right). Elapsed simulation time in minutes is displayed in the lower right corner. Also available in higher quality at `https://youtu.be/ymkaCRvLP6I`.
